# Existence of dynamical low rank approximations for random semi-linear evolutionary equations on the maximal interval

**DOI:** 10.1007/s40072-020-00177-4

**Published:** 2020-08-05

**Authors:** Yoshihito Kazashi, Fabio Nobile

**Affiliations:** Institute of Mathematics, École Polytechnique Fédérale de Lausanne, CSQI, Station 8, CH-1015 Lausanne, Switzerland

**Keywords:** Non-linear evolution equation, Well-posedness, Dynamical low rank approximation, Singular value decomposition

## Abstract

An existence result is presented for the dynamical low rank (DLR) approximation for random semi-linear evolutionary equations. The DLR solution approximates the true solution at each time instant by a linear combination of products of deterministic and stochastic basis functions, both of which evolve over time. A key to our proof is to find a suitable equivalent formulation of the original problem. The so-called Dual Dynamically Orthogonal formulation turns out to be convenient. Based on this formulation, the DLR approximation is recast to an abstract Cauchy problem in a suitable linear space, for which existence and uniqueness of the solution in the maximal interval are established.

## Introduction

This paper is concerned with the existence of solutions of the so called Dynamical Low Rank Method (DLR) [6, 7, 16, 17, 20] to a semi-linear random parabolic evolutionary equation. For a separable ${\mathbb {R}}$-Hilbert space $({\mathcal {H}},\langle \cdot ,\cdot \rangle )$ and a probability space $(\varOmega ,{\mathscr {F}},{\mathbb {P}})$, let $L^{2}(\varOmega ;{\mathcal {H}}):=L_{{\mathbb {P}}}^{2}(\varOmega ;{\mathcal {H}})$ be the Bochner space of equivalence classes of ${\mathcal {H}}$-valued measurable functions on $\varOmega$, with finite second moments. We consider the following equation in $L^{2}(\varOmega ;{\mathcal {H}})$:

1.1 $$\begin{aligned} \frac{\partial u}{\partial t}(t)=\varLambda u(t)+F(u(t)),\quad t>0,\quad \text {with}\ u(0)=u_{0}, \end{aligned}$$

with a closed linear operator $\varLambda :D_{{\mathcal {H}}}(\varLambda )\subset {\mathcal {H}}\rightarrow {\mathcal {H}}$, and a mapping $F:L^{2}(\varOmega ;{\mathcal {H}})\rightarrow L^{2}(\varOmega ;{\mathcal {H}})$, where the domain $D_{{\mathcal {H}}}(\varLambda )$ is dense in ${\mathcal {H}}$. The idea of the DLR approximation is to approximate the solution of (1.1) at each time $t>0$ as a linear combination of products of deterministic and stochastic basis functions, both of which evolve over time: the approximate solution is of the form $u_{S}(t)={\varvec{U}}^{\top }(t){\varvec{Y}}(t)$, for some positive integer $S\in {\mathbb {N}}$ called the rank of the solution, where ${\varvec{U}}(t)=(U_{1}(t),\dots ,U_{S}(t))^{\top }$ are linearly independent in ${\mathcal {H}}$, and ${\varvec{Y}}(t)=(Y_{1}(t),\dots ,Y_{S}(t))^{\top }$ are linearly independent in the space $L^{2}(\varOmega )$ of square-integrable random variables. We note that both bases depend on the temporal variable *t*. This dependence is intended to approximate well, with a fixed (possibly small) rank, the solution of stochastic dynamical systems such as (1.1), whose stochastic and spatial dependence may change significantly in time. Numerical examples and error analysis suggests the method does indeed work well in a certain number of practical applications [17, 20].

A fundamental open question regarding this approach is the unique existence of DLR solutions. The DLR approximation is given as a solution of a system of differential equations, and available approximation results are built upon the assumption that this solution exists, e.g. [6, 16]. Nonetheless, to the best of our knowledge, the existence—let alone the uniqueness—of DLR solutions for an equation of the type (1.1) is not known. In this paper, we will establish a unique existence result.

A difficulty in proving the existence is the fact that the solution propagates in an infinite-dimensional manifold, and that we have an unbounded operator in the equation. Indeed, the DLR equations are derived so that the aforementioned approximation $u_{S}$ keeps the specified form in time, with the fixed rank *S*. By now it is well known that the collection of functions of this form admits an infinite-dimensional manifold structure [5, Sect. 3]. Besides the unbounded operator $\varLambda$, the resulting system of equations involves also a non-linear projection operator onto the tangent space to the manifold, which makes its analysis difficult and non-standard.

Our strategy is to work with a suitable set of parameters describing the manifold, that are elements of a suitable ambient Hilbert space, and invoke results for the evolutionary equations in linear spaces. In utilising such results, the right choice of parametrisation turns out to be crucial. Our choice of parameters leads us to the so-called Dual DO formulation introduced in [17].

A method similar to the DLR approximation is the multi-configuration time-dependent Hartree (MCTDH) method, which has been considered in the context of computational quantum chemistry to approximate a deterministic Schrödinger equation. For the MCTDH method, several existence results have been established, e.g. [2, 12, 13]. The strategy used in these papers, first proposed by Koch and Lubich [13], is to consider a constraint called the gauge condition that is defined by the differential operator in the equation. With their choice of the gauge condition and their specific setting, the differential operator appears outside the projection operator, and this was a crucial step in [2, 12, 13] to apply the standard theory of abstract Cauchy problems. However, as we will see later in Sect. 2.4, the same approach does not work in our setting.

As mentioned above, our strategy is to work with the Dual DO formulation, by which we are able to show that the DLR approximation exists as long as a suitable full rank condition is satisfied. Further, we discuss the extendability of the approximation, beyond the point where we lose the full rankness.

The rest of this paper is organised as follows. In Sect. 2, we introduce the problem under study as well as the Dual DO formulation of the DLR equation. Section 3 introduces a parameter-equation that is equivalent to the Dual DO equations. Then, in Sect. 4 we prove our main result, namely the existence and uniqueness of a DLR solution on the maximal interval. The solution evolves in a manifold up to a maximal time. The solution cannot be continued in this manifold, but we will show that it can be extended in the ambient space, and the resulting continuation will take values in a different manifold with lower rank. Section 5 concludes the paper.

## DLR formulation

In this section, we introduce the setting and recall some facts on the Dynamical Low Rank (DLR) approach that will be needed later.

We detail in Sect. 2.3 the precise assumptions on $\varLambda$, *F* and the initial conditions we will work with. For the moment, we just assume that a solution of (1.1) exists. We note, however, that the existence and uniqueness can be established by standard arguments. For instance, if $\varLambda$ is self-adjoint and satisfies $\langle -\varLambda x,x\rangle \ge 0$ for all $x\in D_{{\mathcal {H}}}(\varLambda )$, by extending the definition of $\varLambda$ to random functions $u\in L^{2}(\varOmega ;{\mathcal {H}})$, where $\varLambda :D(\varLambda )\subset L^{2}(\varOmega ;{\mathcal {H}}) \rightarrow L^{2}(\varOmega ;{\mathcal {H}})$ is applied pointwise in $\varOmega$, we have that $\varLambda$ is densely defined, closed, and satisfies

$$\begin{aligned} {\mathbb {E}}[\langle -\varLambda v,v\rangle ]\ge 0\quad \text { for all }v\in D(\varLambda )\subset L^{2}(\varOmega ;{\mathcal {H}}). \end{aligned}$$

Together with a local Lipschitz continuity of *F*, existence of solutions can be established by invoking a standard theory of semi-linear evolution equations, see for example [18, 21].

We define an element $u_{S}\in L^{2}(\varOmega ;{\mathcal {H}})$ to be an *S*-*rank random field* if $u_{S}$ can be expressed as a linear combination of *S* (and not less than *S*) linearly independent elements of ${\mathcal {H}}$, and *S* (and not less than *S*) linearly independent elements of $L^{2}(\varOmega )$. Further, we let ${\hat{M}}_{S}\subset L^{2}(\varOmega ;{\mathcal {H}})$ be the collection of all the *S*-rank random fields:

$$\begin{aligned} {\hat{M}}_{S} \!:=\Bigg \{u_{S}\!=\!\sum _{j=1}^{S}U_{i}Y_{i} \,\Bigg |\, \begin{array}{rl} \{U_{j}\}_{j=1}^{S}&{}\!\!\text { is linear independent in }{\mathcal {H}}\\ \{Y_{j}\}_{j=1}^{S}&{}\!\!\text { is linear independent in }L^{2}(\varOmega )\end{array} \Bigg \}. \end{aligned}$$

It is known that ${\hat{M}}_{S}$ can be equipped with a differentiable manifold structure, see [5, 17]. The idea behind the DLR approach is to approximate the curve $t\mapsto u(t)\in L^2(\varOmega ;{\mathcal {H}})$ defined by the solution of the Eq. (1.1) by a curve $t\mapsto u_{S}(t)\in {\hat{M}}_{S}$ given as a solution of the following problem: find $u_{S}\in {\hat{M}}_{S}$ such that $u_{S}(0)={u_{0S}}\in {\hat{M}}_{S}$, a suitable approximation of $u_0$ in ${\hat{M}}_{S}$, and for (almost) all $t>0$ we have $\frac{\partial u_{S}}{\partial t}(t)-(\varLambda u_{S}(t)+F(u_{S}(t)))\in L^{2}(\varOmega ;{\mathcal {H}})$ and

2.1 $$\begin{aligned} {\mathbb {E}}\Big [\Big \langle \frac{\partial u_{S}}{\partial t}(t)-(\varLambda u_{S}(t)+F(u_{S}(t))),v\Big \rangle \Big ]=0,\;\text { for all }v\in T_{u_{S}(t)}{\hat{M}}_{S}, \end{aligned}$$

where $T_{u_{S}(t)}{\hat{M}}_{S}{\subset L^{2}(\varOmega ;{\mathcal {H}})}$ is the tangent space of ${\hat{M}}_{S}$ at $u_{S}(t)$, and ${\mathbb {E}}[\cdot ]$ denotes expectation with respect to the underlying probability measure ${\mathbb {P}}$.

In this paper, we search for the solution in the same set as ${\hat{M}}_{S}$ but with a different parametrisation that is easier to work with. The set

2.2 $$\begin{aligned} {M}_{S}:=\Bigg \{u_{S}\!=\!\sum _{j=1}^{S}U_{i}Y_{i} \,\Bigg |\, \begin{array}{rl} \{U_{j}\}_{j=1}^{S}&{}\!\!\text { is linear independent in }{\mathcal {H}}\\ \{Y_{j}\}_{j=1}^{S}&{}\!\!\text { is orthonormal in }L^{2}(\varOmega )\end{array} \Bigg \} \end{aligned}$$

is the same subset of $L^{2}(\varOmega ;{\mathcal {H}})$ as ${\hat{M}}_{S}$, and thus the above problem is equivalent when we seek solutions in $M_{S}$ instead of ${\hat{M}}_{S}$. This leads us to the so-called Dual Dynamically Orthogonal (DO) formulation of the problem (2.1).

For $u_{S}={\varvec{U}}^{\top }{\varvec{Y}}\in M_{S}$, define the operator ${\mathscr {P}}_{u_{S}}:L^{2}(\varOmega ;{\mathcal {H}})\rightarrow L^{2}(\varOmega ;{\mathcal {H}})$ by

$$\begin{aligned} {\mathscr {P}}_{u_{S}}:=P_{{\varvec{U}}}+P_{{\varvec{Y}}}-P_{{\varvec{U}}}P_{{\varvec{Y}}}, \end{aligned}$$

where, for an arbitrary ${\mathcal {H}}$-orthonormal basis $\{\phi _{j}\}_{j=1}^{S}\subset {\mathcal {H}}$ of $\mathrm {span}_{{\mathbb {R}}}\{\{U_{j}\}_{j=1}^{S}\}$ the operator $P_{{\varvec{U}}}:L^{2}(\varOmega ;{\mathcal {H}})\rightarrow L^{2}(\varOmega ;{\mathcal {H}})$ is defined by $P_{{\varvec{U}}}f=\sum _{j=1}^{S}\langle f,\phi _{j}\rangle \phi _{j}$ for $f\in L^{2}(\varOmega ;{\mathcal {H}}),$ and moreover, for an arbitrary $L^{2}(\varOmega )$-orthonormal basis $\{\psi _{j}\}_{j=1}^{S}\subset L^{2}(\varOmega )$ of $\mathrm {span}_{{\mathbb {R}}}\{\{Y_{j}\}_{j=1}^{S}\}$ the operator $P_{{\varvec{Y}}}:L^{2}(\varOmega ;{\mathcal {H}})\rightarrow L^{2}(\varOmega ;{\mathcal {H}})$ is defined by

2.3 $$\begin{aligned} P_{{\varvec{Y}}}f=\sum _{j=1}^{S}{\mathbb {E}}[f\psi _{j}]\psi _{j}\ \text {for }f\in L^{2}(\varOmega ;{\mathcal {H}}). \end{aligned}$$

This operator ${\mathscr {P}}_{u_{S}}$ turns out to be the $L^{2}(\varOmega ;{\mathcal {H}})$-orthogonal projection to the tangent space $T_{u_{S}}M_{S}$ at $u_{S}={\varvec{U}}{}^{\top }{\varvec{Y}}$, see [16, Proposition 3.3] together with [4]. Note that ${\mathscr {P}}_{u_{S}}$ is independent of the choice of the representation of $u_{S}$.

Using the above definitions, the problem we consider, equivalent to (2.1), can be formulated as follows:

### Problem 1

Find ${t\mapsto u_{S}(t)}\in M_{S}$ such that $u_{S}(0)={u_{0S}}\in M_{S}$ and for $t>0$ we have

2.4 $$\begin{aligned} \frac{\partial u_{S}}{\partial t}(t)={\mathscr {P}}_{u_{S}(t)}(\varLambda u_{S}(t)+F(u_{S}(t))). \end{aligned}$$

We consider two notions of solutions: the strong and classical solution.

### Definition 2.1

(*Strong DLR solution*) A function $u_{S}:[0,T]\rightarrow M_{S}\subset L^{2}(\varOmega ;{\mathcal {H}})$ is called a *strong DLR solution* if $u_{S}(0)={u_{0S}}\in M_{S}$, $u_{S}$ is absolutely continuous on [0, *T*], and (2.4) is satisfied a.e. on [0, *T*]. Further, we call $u_S$ a *strong DLR solution on* [0, *T*) if it is a strong DLR solution on any subinterval $[0,T']\subset [0,T)$.

### Definition 2.2

(*Classical DLR solution*) A function $u_{S}:[0,T]\rightarrow M_{S}\subset L^{2}(\varOmega ;{\mathcal {H}})$ is called a *classical DLR solution* on [0, *T*] if $u_{S}(0)={u_{0S}}\in M_{S}$, $u_{S}$ is absolutely continuous on [0, *T*], continuously differentiable on (0, *T*], $u_{S}{\in D(\varLambda )}$ for $t\in {(0,T]}$, and (2.4) is satisfied on (0, *T*]. Further, we call $u_S$ a *classical DLR solution on* [0, *T*) when it is a classical DLR solution on any subinterval $[0,T']\subset [0,T)$.

### Dual DO formulation

Our aim is to establish the unique existence of a DLR solution. Our strategy is to choose a suitable parametrisation of ${M}_{S}$, and work in a linear space which the parameters belong to. For the parametrisation, we will choose the one proposed in [17], which results in a formulation of (2.4) called Dual DO, where we seek an approximate solution of the form $u_{S}(t)={\varvec{U}}^{\top }(t){\varvec{Y}}(t)\in M_{S}$ for any [0, *T*]. Here, the parameter $({\varvec{U}}(t),{\varvec{Y}}(t))\in [{\mathcal {H}}]^{S}\times [L^{2}(\varOmega )]^{S}$ is a solution to the following problem:

1. ${\varvec{U}}(t)=(U_{1}(t),\dots ,U_{S}(t))^{\top }$ are linearly independent in ${\mathcal {H}}$ for any $t\in [0,T]$;
2. ${\varvec{Y}}(t)=(Y_{1}(t),\dots ,Y_{S}(t))^{\top }$ are orthonormal in $L^{2}(\varOmega )$ for any $t\in [0,T]$, and satisfy the so-called gauge condition: for any $t\in (0,T)$,
  $$\begin{aligned} {\mathbb {E}}\bigg [\frac{\partial Y_{j}}{\partial t}Y_{k}\bigg ]=0\ \text {for}\ j,k=1,\dotsc ,S,\text { equivalently, }{\mathbb {E}}\bigg [\frac{\partial {\varvec{Y}}}{\partial t}{\varvec{Y}}^{\top }\bigg ]=0\in {\mathbb {R}}^{S\times S}; \end{aligned}$$
3. $({\varvec{U}},{\varvec{Y}})$ satisfies the equation
  2.5 $$\begin{aligned} \left\{ \begin{array}{rl} \frac{\partial }{\partial t}{\varvec{U}}&{} ={\mathbb {E}}[{\mathcal {L}}(u_S){\varvec{Y}}]\\ Z_{{\varvec{U}}}\frac{\partial }{\partial t}{\varvec{Y}} &{} =(I-P_{{\varvec{Y}}})[\langle {\mathcal {L}}(u_S),{\varvec{U}}\rangle ], \end{array}\right. \end{aligned}$$
  where ${\mathcal {L}}:={\varLambda +F}$, $P_{{\varvec{Y}}}$ is as in (2.3), and $Z_{{\varvec{U}}}={(\langle U_{j},U_{k}\rangle )_{j,k=1,\dotsc ,S}}\in {\mathbb {R}}^{S\times S}$ is the Gram matrix defined by ${\varvec{U}}$;
4. $({\varvec{U}},{\varvec{Y}})$ satisfies the initial condition $({\varvec{U}}(0),{\varvec{Y}}(0))=({\varvec{U}}_{0},{\varvec{Y}}_{0})$ for some $({\varvec{U}}_{0},{\varvec{Y}}_{0})\in [{\mathcal {H}}]^{S}\times [L^{2}(\varOmega )]^{S}$ such that ${\varvec{U}}_{0}^{\top }{\varvec{Y}}_{0}{=u_{0S}}\in M_{S}$.

Noting that, since the operator $\varLambda$ is deterministic and linear, we have

$$\begin{aligned} P_{{\varvec{Y}}}(\langle \varLambda (u_{S}),{\varvec{U}}\rangle ) =P_{{\varvec{Y}}}(\langle \varLambda ({\varvec{U}}^{\top }){\varvec{Y}},{\varvec{U}}\rangle ) =\langle \varLambda (u_{S}),{\varvec{U}}\rangle \end{aligned}$$

and ${\mathbb {E}}[\varLambda (u_{S}){\varvec{Y}}^{\top }] =\varLambda ({\varvec{U}}^{{\top }}){\mathbb {E}}[{\varvec{Y}}{\varvec{Y}}^{\top }] =\varLambda ({\varvec{U}}^\top )$, the Eq. (2.5) reads

2.6 $$\begin{aligned} \left\{ \begin{array}{rll} \frac{\partial }{\partial t}{\varvec{U}} &{} =\varLambda ({\varvec{U}})+{\mathbb {E}}\big [F({\varvec{U}}^{\top }{\varvec{Y}}){\varvec{Y}}\big ] &{} =:\varLambda ({\varvec{U}})+G_{1}({\varvec{Y}})({\varvec{U}})\\ \frac{\partial }{\partial t}{\varvec{Y}} &{} =(I-P_{{\varvec{Y}}})(\langle F({\varvec{U}}^{\top }{\varvec{Y}}),Z_{{\varvec{U}}}^{-1}{\varvec{U}}\rangle ) &{} =:G_{2}({\varvec{U}})({\varvec{Y}}). \end{array}\right. \end{aligned}$$

We define two notions of solutions to the initial value problem of (2.6) that correspond to those of the original problem as in Definitions 2.1–2.2.

#### Definition 2.3

(*Strong dual DO solution*) A function $({\varvec{U}},{\varvec{Y}}):[0,T]\rightarrow [{\mathcal {H}}]^{S}\times [L^{2}(\varOmega )]^{S}$ is called a *strong Dual DO solution * if it satisfies the following conditions:

1. $({\varvec{U}}(0),{\varvec{Y}}(0))=({\varvec{U}}_{0},{\varvec{Y}}_{0})$ for some $({\varvec{U}}_{0},{\varvec{Y}}_{0})\in [{\mathcal {H}}]^{S}\times [L^{2}(\varOmega )]^{S}$ such that ${u_{0S}}={\varvec{U}}_{0}^{\top }{\varvec{Y}}_{0}\in M_{S}$;
2. $({\varvec{U}},{\varvec{Y}})$ satisfies the Eq. (2.6) a.e. on [0, *T*];
3. the curve $t\mapsto {\varvec{U}}(t)\in [{\mathcal {H}}]^{S}$ is absolutely continuous on [0, *T*];
4. the curve $t\mapsto {\varvec{Y}}(t)\in [L^{2}(\varOmega )]^{S}$ is absolutely continuous on [0, *T*];
5. $\{U_{j}(t)\}_{j=1}^{S}$ is linear independent in ${\mathcal {H}}$ for almost every $t\in [0,T]$; and
6. $\{Y_{j}(t)\}_{j=1}^{S}$ is orthonormal in $L^{2}(\varOmega )$ for almost every $t\in [0,T]$.

Notice, in particular, that the condition 5 above implies that the matrix $Z_{{\varvec{U}}}$ is invertible for almost every $t\in [0,T]$. Further, from (2.6) we necessarily have

2.7 $$\begin{aligned} {\mathbb {E}}\Big [\Big (\frac{\partial }{\partial t}{\varvec{Y}}\Big ){\varvec{Y}}^{\top }\Big ]={\mathbb {E}}\Big [\langle F({\varvec{U}}^{\top }{\varvec{Y}}),Z_{{\varvec{U}}}^{-1}{\varvec{U}}\rangle (I-P_{{\varvec{Y}}}){\varvec{Y}}^{\top }\Big ]=0. \end{aligned}$$

#### Definition 2.4

(*Classical dual DO solution*) A function $({\varvec{U}},{\varvec{Y}}):[0,T]\rightarrow [{\mathcal {H}}]^{S}\times [L^{2}(\varOmega )]^{S}$ is called a *classical Dual DO solution * if it satisfies the following conditions:

1. $({\varvec{U}}(0),{\varvec{Y}}(0))=({\varvec{U}}_{0},{\varvec{Y}}_{0})$ for some $({\varvec{U}}_{0},{\varvec{Y}}_{0})\in [{\mathcal {H}}]^{S}\times [L^{2}(\varOmega )]^{S}$ such that ${u_{0S}}={\varvec{U}}_{0}^{\top }{\varvec{Y}}_{0}\in M_{S}$;
2. $({\varvec{U}},{\varvec{Y}})$ satisfies the Eq. (2.6) on (0, *T*];
3. the curve $t\mapsto {\varvec{U}}(t)\in [{\mathcal {H}}]^{S}$ is absolutely continuous on [0, *T*], continuously differentiable on (0, *T*];
4. the curve $t\mapsto {\varvec{Y}}(t)\in [L^{2}(\varOmega )]^{S}$ is absolutely continuous on [0, *T*], continuously differentiable on (0, *T*];
5. $$U_{j}(t)\in D_{{\mathcal {H}}}(\varLambda )$$ for any $$t\in {(0,T]}$$, $$j=1,\dots ,S$$;
6. $\{U_{j}(t)\}_{j=1}^{S}$ is linear independent in ${\mathcal {H}}$ for any $t\in [0,T]$;
7. $\{Y_{j}(t)\}_{j=1}^{S}$ is orthonormal in $L^{2}(\varOmega )$ for any $t\in [0,T]$.

#### Definition 2.5

If $({\varvec{U}},{\varvec{Y}}):[0,T)\rightarrow [{\mathcal {H}}]^{S}\times [L^{2}(\varOmega )]^{S}$ is a strong (resp. classical) Dual DO solution on all subintervals $[0,T']\subset [0,T)$, then we call $({\varvec{U}},{\varvec{Y}})$ a *strong (resp. classical) Dual DO solution on* [0, *T*).

### Equivalence with the original formulation

In this section, we establish the equivalence of the original equation (2.4) and the Dual DO formulation as in Definitions 2.3–2.4. Our first step is to show that if a DLR solution is given, then there exists a unique corresponding Dual DO solution, see Lemma 2.5.

We will need a proposition which states that if $t\mapsto u_S(t)\in M_S\subset L^{2}(\varOmega ;{\mathcal {H}})$ is differentiable, then there exists a differentiable parametrisation.

We start with the following lemma.

#### Lemma 2.1

Let $u_{S}\in M_{S}\subset L^{2}(\varOmega ;{\mathcal {H}})$ be given. Then, with some $\{{\tilde{V}}_{j}\}_{j=1}^{S}$ and $\{W_{j}\}_{j=1}^{S}$ orthonormal in ${\mathcal {H}}$ and $L^{2}(\varOmega )$, respectively, and $\sigma _{j}>0$, $j=1,\dots ,S$, we have

$$\begin{aligned} u_{S}=\sum _{j=1}^{S}\sigma _{j}{\tilde{V}}_{j}W_{j}. \end{aligned}$$

Moreover, such $\sigma _{j}>0$ is unique in the following sense: for any other representation $u_{S}=\sum _{j=1}^{S}\sigma '_{j}{\tilde{V}}'_{j}W'_{j}$ with $\{{\tilde{V}}_{j}'\}_{j=1}^{S}$ and $\{W_{j}'\}_{j=1}^{S}$ orthonormal, upon relabelling if necessary, we have $\sigma '_{j}=\sigma {}_{j}$, $j=1,\dots ,S$. Furthermore, if $[0,T]\ni t\mapsto u_{S}(t)\in M_{S}\subset L^{2}(\varOmega ;{\mathcal {H}})$ is continuous, then the corresponding values $\{\sigma _{j}(t)\}_{j=1}^{S}$ satisfy

2.8 $$\begin{aligned} 0<\min _{j=1,\dots ,S}\inf _{t\in [0,T]}\sigma _{j}(t)\qquad \text {and}\qquad \max _{j=1,\dots ,S}\sup _{t\in [0,T]}\sigma _{j}(t)<\infty . \end{aligned}$$

#### Proof

The linear operator $K=K(u_{S})$ defined by $L^{2}(\varOmega )\ni w\mapsto Kw:={\mathbb {E}}[u_{S}w]\in {\mathcal {H}}$ is a finite-rank operator with rank *S*, with the image being independent of the representation of $u_{S}={\varvec{U}}^{\top }{\varvec{Y}}\in M_{S}$.

Thus, with some $\{{\tilde{V}}_{j}\}_{j=1}^{S}$ and $\{W_{j}\}_{j=1}^{S}$ orthonormal in ${\mathcal {H}}$ and $L^{2}(\varOmega )$, respectively, *K* admits the canonical decomposition

$$\begin{aligned} Kw=\sum _{j=1}^{S}\sigma _{j}{\mathbb {E}}[wW_{j}]{\tilde{V}}_{j}, \end{aligned}$$

with singular values $\sigma _{j}=\sigma _{j}(K)>0$, $j=1,\dots ,S$, see e.g. [9, Sects. III.4.3 and V.2.3]. Observe that, if we have another representation $u_{S}=\sum _{j=1}^{S}\sigma '_{j}{\tilde{V}}'_{j}W'_{j}$, then upon relabelling if necessary we must have $\sigma '_{j}=\sigma _{j}$.

To show (2.8), relabel $\{\sigma _{j}(t)\}_{j=1}^{S}$ in the non-decreasing order and denote it by $(\alpha _{j}(t))_{j=1}^{S}$. Then, for any $t\in [0,T]$ and $h\in {\mathbb {R}}$ such that $t+h\in [0,T]$ we have $|\alpha _{j}(t+h)-\alpha _{j}(t)| \le \Vert K(u_{S}(t+h))-K(u_{S}(t))\Vert _{L^{2}(\varOmega )\rightarrow {\mathcal {H}}}\text { for }j=1,\dots ,S$, see for example [19, Proposition II.7.6 and Theorem IV.2.2]. But we have

$$\begin{aligned} \Vert K(u_{S}(t+h))w-K(u_{S}(t))w\Vert _{{\mathcal {H}}}&\le \Big ({\mathbb {E}}[\Vert u_{S}(t+h)-u_{S}(t)\Vert _{{\mathcal {H}}}^{2}]\Big )^{1/2}\Vert w\Vert _{L^{2}(\varOmega )}, \end{aligned}$$

for any $w\in L^{2}(\varOmega )$, and thus the continuity of $t\mapsto u_{S}(t)$ implies that $\alpha _{j}$ is continuous on [0, *T*]. Now, since *K* is of rank *S* for any $t\in [0,T]$, we have $\alpha _{j}(t)>0$ for any $t\in [0,T]$. Hence, for $j=1,\dots ,S$ we have

$$\begin{aligned} \inf _{t\in [0,T]}\sigma _{j}(t)\ge \min _{t\in [0,T]}\alpha _{1}(t)>0. \end{aligned}$$

Similarly, $\sup _{t\in [0,T]}\sigma _{j}(t)\le \max _{t\in [0,T]}\alpha _{S}(t)$, which completes the proof. $\square$

#### Proposition 2.2

Suppose that $[0,T]\ni t\mapsto u_{S}(t)\in M_{S}\subset L^{2}(\varOmega ;{\mathcal {H}})$ is absolutely continuous. Then, there exist $t\mapsto {\tilde{V}}_{j}(t)\in {\mathcal {H}}$, $t\mapsto \varSigma (t)\in {\mathbb {R}}^{S\times S}$, and $t\mapsto W_{j}(t)\in L^{2}(\varOmega )$, $j=1,\dots ,S$ such that

$$\begin{aligned} u_{S}(t)= \tilde{{\varvec{V}}}(t)^\top \varSigma (t){\varvec{W}}(t) \qquad \text {for all }t\in [0,T]; \end{aligned}$$

$\{{\tilde{V}}_{j}(t)\}_{j=1}^{S}$ and $\{W_{j}(t)\}_{j=1}^{S}$ are orthonormal in ${\mathcal {H}}$ and in $L^{2}(\varOmega )$, respectively; $\varSigma (t)$ is full rank; the curves $t\mapsto \varSigma (t)\in {\mathbb {R}}^{S\times S}$, $t\mapsto {\tilde{V}}_{j}(t)\in {\mathcal {H}}$, and $t\mapsto W_{j}(t)\in L^{2}(\varOmega )$, $j=1,\dots ,S$ are absolutely continuous on [0, *T*]. Moreover, if $u_{S}$ is continuously differentiable on (0, *T*], then ${\tilde{V}}_{j}$, $\varSigma$, and $W_{j}$ are continuously differentiable on (0, *T*].

In particular, $u_{S}(t)$ admits a representation $u_{S}={\varvec{V}}^{\top }{\varvec{W}}$ in $M_{S}$ with ${\varvec{V}}^{\top }=\tilde{{\varvec{V}}}^{\top }\varSigma$, with the specified smoothness.

To show Proposition 2.2, we will use an argument similar to what we will see in Sect. 4 below. Thus, we will defer the proof to Sect. 4.

Parametrisation of $M_{S}$ is determined by parameters up to a unique orthogonal matrix.

#### Lemma 2.3

Let $v_{S}\in M_{S}$ be given. Suppose that $v_{S}$ admits two representations $v_{S}={\varvec{V}}^{\top }{\varvec{W}}=\tilde{{\varvec{V}}}^{\top }\tilde{{\varvec{W}}}\in M_{S}$ with some $({\varvec{V}},{\varvec{W}}),\ (\tilde{{\varvec{V}}},\tilde{{\varvec{W}}})\in [H]^{S}\times [L^{2}(\varOmega )]^{S}$ satisfying the linear independence and orthonormality conditions as in (2.2). Then, we have $(\tilde{{\varvec{V}}},\tilde{{\varvec{W}}})=(\varTheta {}^{\top }{\varvec{V}},\varTheta {}^{\top }{\varvec{W}})$ for a unique $\varTheta \in O(S)$.

#### Proof

From $\tilde{{\varvec{V}}}^{\top }\tilde{{\varvec{W}}}={\varvec{V}}{}^{\top }{\varvec{W}}$, we have

$$\begin{aligned} \tilde{{\varvec{W}}}=(\langle \tilde{{\varvec{V}}},\tilde{{\varvec{V}}}{}^{\top }\rangle )^{-1}\langle \tilde{{\varvec{V}}},{\varvec{V}}{}^{\top }\rangle {\varvec{W}}=:\varTheta {}^{\top }{\varvec{W}}, \end{aligned}$$

so that $\tilde{{\varvec{W}}}\tilde{{\varvec{W}}}{}^{\top }=\varTheta {}^{\top }{\varvec{W}}{\varvec{W}}{}^{\top }\varTheta$. From the $L^{2}(\varOmega )$-orthonormality of $\tilde{{\varvec{W}}}$ and ${\varvec{W}}$, taking the expectation of both sides we conclude that $\varTheta$ is an orthogonal matrix. The uniqueness is easy to see. $\square$

The above lemma implies the following corollary, which states that if both a DLR solution $u_{S}$ and a Dual DO solution $({\varvec{U}},{\varvec{Y}})$ exist, and if further the DLR solution is unique, then $({\varvec{U}},{\varvec{Y}})$ is determined by $u_{S}$ up to a unique orthogonal matrix. We stress that the next corollary does not guarantee the uniqueness of the Dual DO solution.

#### Corollary 2.4

Suppose that a strong DLR solution $u_{S}(t)\in M_{S}$, $t\in [0,T]$ uniquely exists. Let $({\varvec{V}}(t),{\varvec{W}}(t))\in [H]^{S}\times [L^{2}(\varOmega )]^{S}$ be any representation of $u_S(t)$, namely $u_{S}(t)={\varvec{V}}(t)^{\top }{\varvec{W}}(t)$, satisfying the linear independence and orthonormality conditions defined in (2.2). Furthermore, suppose that a Dual DO solution $({\varvec{U}}(t),{\varvec{Y}}(t))$ exists in the strong sense. Then, we have

2.9 $$\begin{aligned} ({\varvec{U}}(t),{\varvec{Y}}(t))=(\varTheta (t)^{\top }{\varvec{V}}(t),\varTheta (t)^{\top }{\varvec{W}}(t)), \end{aligned}$$

for a unique $\varTheta (t)\in O(S)$. In words, if a Dual DO solution $({\varvec{U}},{\varvec{Y}})$ exists, then it must be of the form $(\varTheta ^{\top }{\varvec{V}},\varTheta ^{\top }{\varvec{W}})$ with an arbitrarily chosen representation ${\varvec{V}}^{\top }{\varvec{W}}$ of $u_{S}$ and the corresponding unique orthogonal matrix $\varTheta$.

#### Proof

We first show that the function ${\hat{u}}_{S}:={\varvec{U}}(t)^{\top }{\varvec{Y}}(t)\in M_{S}$ satisfies the original equation (2.4). Since $({\varvec{U}}(t),{\varvec{Y}}(t))$ is a Dual DO solution in the strong sense, from (2.6) a.e. on [0, *T*] we have

$$\begin{aligned} \frac{\mathrm {d}}{\mathrm {d}t}{\hat{u}}_{S}&=\frac{\mathrm {d}}{\mathrm {d}t}{\varvec{U}}^{\top }{\varvec{Y}}+{\varvec{U}}^{\top }\frac{\mathrm {d}}{\mathrm {d}t}{\varvec{Y}}\\ {}&=\varLambda ({\hat{u}}_{S})+P_{{\varvec{Y}}}(F({\hat{u}}_{S}))+(I-P_{{\varvec{Y}}})P_{{\varvec{U}}}(F({\hat{u}}_{S}))\in L^{2}(\varOmega ;{\mathcal {H}}). \end{aligned}$$

Now, notice that $P_{{\varvec{Y}}}\varLambda ({\hat{u}}_{S})=\varLambda ({\hat{u}}_{S})$ and thus $(P_{{\varvec{U}}}-P_{{\varvec{U}}}P_{{\varvec{Y}}})\varLambda ({\hat{u}}_{S})=0$. Together with $P_{{\varvec{U}}}P_{{\varvec{Y}}}=P_{{\varvec{Y}}}P_{{\varvec{U}}}$ we obtain $\frac{\mathrm {d}}{\mathrm {d}t}{\hat{u}}_{S}=(P_{{\varvec{Y}}}+(P_{{\varvec{U}}}-P_{{\varvec{U}}}P_{{\varvec{Y}}}))\varLambda ({\hat{u}}_{S})+(P_{{\varvec{Y}}}+P_{{\varvec{U}}}-P_{{\varvec{U}}}P_{{\varvec{Y}}})F({\hat{u}}_{S})$, which is (2.4).

Then, from the uniqueness of the DLR solution we have ${\varvec{V}}(t)^{\top }{\varvec{W}}(t) ={\varvec{U}}(t)^{\top }{\varvec{Y}}(t)$. Thus, in view of Lemma 2.3 the statement follows. $\square$

In Corollary 2.4, we assumed the existence of both the DLR solution and the Dual DO solution, and deduced the existence of a unique orthogonal matrix. The following lemma shows that the existence of a Dual DO solution is implied by the existence of a DLR solution.

The proof is inspired by [11, Proof of Proposition II.3.1].

#### Lemma 2.5

Let a strong DLR solution $[0,T]\ni t\mapsto u_{S}(t)\in M_{S}\subset L^{2}(\varOmega ;{\mathcal {H}})$ with $u_S(0)=u_{0S}\in M_S$ be given. Let $({\varvec{V}}(0),{\varvec{W}}(0))\in [{\mathcal {H}}]^{S}\times [L^{2}(\varOmega )]^{S}$ be such that ${\varvec{V}}(0)^\top {\varvec{W}}(0)=u_{0S}$. Then, there exists a strong Dual DO solution $({\varvec{U}},{\varvec{Y}})$ with the initial condition $({\varvec{V}}(0),{\varvec{W}}(0))\in [{\mathcal {H}}]^{S}\times [L^{2}(\varOmega )]^{S}$. Further, $({\varvec{U}},{\varvec{Y}})$ is the unique Dual DO solution such that $u_{S}(t)={\varvec{U}}(t)^{\top }{\varvec{Y}}(t)$ for all $t\in [0,T]$.

#### Proof

From Proposition 2.2, there exists a curve $t\mapsto {(\tilde{{\varvec{V}}}(t),\tilde{{\varvec{W}}}(t))}\in [{\mathcal {H}}]^{S}\times [L^{2}(\varOmega )]^{S}$ such that $u_{S}(t)={\tilde{{\varvec{V}}}(t)^{\top }\tilde{{\varvec{W}}}(t)}$ for all $t\in [0,T]$; $\{{{\tilde{V}}_{j}}\}_{j=1}^{S}$ is linear independent in ${\mathcal {H}}$; $\{{{\tilde{W}}_{j}}\}_{j=1}^{S}$ is orthonormal in $L^{2}(\varOmega )$; $t\mapsto {\tilde{{\varvec{V}}}}(t)\in [{\mathcal {H}}]^{S}$ and $t\mapsto {\tilde{{\varvec{W}}}}(t)\in L^{2}(\varOmega )$ are absolutely continuous on [0, *T*]. In general, $\tilde{{\varvec{V}}}(0)\ne {\varvec{V}}(0)$ and $\tilde{{\varvec{W}}}(0)\ne {\varvec{W}}(0)$, but from Lemma 2.3, one can find a unique orthogonal matrix ${\varXi }$ such that

$$\begin{aligned} \varXi \tilde{{\varvec{V}}}(0)={\varvec{V}}(0)\ \text { and }\ \varXi \tilde{{\varvec{W}}}(0)={\varvec{W}}(0). \end{aligned}$$

Now, let $\varXi \tilde{{\varvec{V}}}(t):={\varvec{V}}(t)$ and $\varXi \tilde{{\varvec{W}}}(t):={\varvec{W}}(t)$, so that $u_S(t)={\varvec{V}}^\top (t){\varvec{W}}(t)$. Notice that $t\mapsto {\varvec{V}}(t)$ and $t\mapsto {\varvec{W}}(t)$ are absolutely continuous. From Corollary 2.4, if the Dual DO solution $({\varvec{U}}(t),{\varvec{Y}}(t))$ exists then we necessarily have

2.10 $$\begin{aligned} ({\varvec{U}}(t),\!{\varvec{Y}}(t))=(\varTheta (t)^{\top }{\varvec{V}}(t),\varTheta (t)^{\top }{\varvec{W}}(t)), \text { for a unique }\varTheta (t)\in O(S). \end{aligned}$$

We show that such $\varTheta (t)$, i.e. an orthogonal matrix $\varTheta (t)$ for which the pair $(\varTheta ^{\top }{\varvec{V}},\varTheta ^{\top }{\varvec{W}})$ is a Dual DO solution, uniquely exists. Note that again from Corollary 2.4, it suffices to consider an arbitrarily fixed representation $({\varvec{V}},{\varvec{W}})$. We will obtain $\varTheta$ as a solution of an ordinary differential equation we will now derive. If $({\varvec{U}},{\varvec{Y}})$ is a Dual DO solution, then (2.10) implies

$$\begin{aligned} (\dot{{\varvec{U}}}(t),\dot{{\varvec{Y}}}(t))=\Big (\frac{\mathrm {d}}{\mathrm {d}t}\big (\varTheta (t)^{\top }{\varvec{V}}(t)\big ),{\dot{\varTheta }}(t)^{\top }{\varvec{W}}(t)+\varTheta (t)^{\top }\dot{{\varvec{W}}}(t)\Big ), \end{aligned}$$

and from (2.7) we must have

$$\begin{aligned} 0={\mathbb {E}}[{\varvec{Y}}(t)\dot{{\varvec{Y}}}(t)^{\top }]&={\mathbb {E}}\big [\varTheta (t)^{\top }{\varvec{W}}(t)\big ({\dot{\varTheta }}(t)^{\top }{\varvec{W}}(t)+\varTheta (t)^{\top }\dot{{\varvec{W}}}(t)\big )^{\top }\big ]\\&=\varTheta (t)^{\top }{\mathbb {E}}[{\varvec{W}}(t){\varvec{W}}(t)^{\top }]{\dot{\varTheta }}(t)+\varTheta (t)^{\top }{\mathbb {E}}[{\varvec{W}}(t)\dot{{\varvec{W}}}(t)^{\top }]\varTheta (t)\\&=\varTheta (t)^{\top }\big ({\dot{\varTheta }}(t)+{\mathbb {E}}[{\varvec{W}}(t)\dot{{\varvec{W}}}(t)^{\top }]\varTheta (t)\big ), \end{aligned}$$

where in the last line we used ${\mathbb {E}}[{\varvec{W}}(t){\varvec{W}}(t)^{\top }]=I$. Using the orthonormality of $\varTheta$ yields the equation

2.11 $$\begin{aligned} {\dot{\varTheta }}(t)=-{\mathbb {E}}[{\varvec{W}}(t)\dot{{\varvec{W}}}(t)^{\top }]\varTheta (t),\quad t\in (0,T)\ \text { with }\varTheta (0)=I. \end{aligned}$$

Now, from the assumptions we have

2.12 $$\begin{aligned} \int _{0}^{T}\!\Vert {\mathbb {E}}[{\varvec{W}}(t)\dot{{\varvec{W}}}(t)^{\top }]\Vert _{\mathrm {F}}\mathrm {d}t\le \!\sup _{s\in [0,T]}\Vert {\varvec{W}}(s)\Vert _{[L^{2}(\varOmega )]^{S}}\int _{0}^{T}\Vert \dot{{\varvec{W}}}(t)\Vert _{[L^{2}(\varOmega )]^{S}}\mathrm {d}t<\infty ,\nonumber \\ \end{aligned}$$

where $\Vert \cdot \Vert _{\mathrm {F}}$ denotes the Frobenius norm, and thus $-{\mathbb {E}}[{\varvec{W}}(\cdot )\dot{{\varvec{W}}}(\cdot )^{\top }]\in {\mathbb {R}}^{S\times S}$ is integrable on (0, *T*). Thus, from a standard fixed-point argument we obtain that a solution $\varTheta \in C([0,T];{\mathbb {R}}^{S\times S})$ of the integral equation $\varTheta (t)=I-\int _{0}^{t}{\mathbb {E}}[{\varvec{W}}(s)\dot{{\varvec{W}}}(s)^{\top }]\varTheta (s)\mathrm {d}s$, $t\in [0,T]$ uniquely exists in $C([0,T];{\mathbb {R}}^{S\times S})$. The solution $\varTheta$ thus obtained is absolutely continuous on [0, *T*], and satisfies (2.11) a.e. on (0, *T*) [14, Theorem 1.17]. Moreover, we have $\varTheta (t)\in O(S)$ for all $t\in [0,T]$: for a.e. on [0, *T*]

$$\begin{aligned} \frac{\mathrm {d}}{\mathrm {d}t}(\varTheta ^{\top }\varTheta )&=-\varTheta (t)^{\top }({\mathbb {E}}[{\varvec{W}}(t)\dot{{\varvec{W}}}(t)^{\top }])^{\top }\varTheta (t)-\varTheta (t)^{\top }{\mathbb {E}}[{\varvec{W}}(t)\dot{{\varvec{W}}}(t)^{\top }]\varTheta (t)\\&=\varTheta (t)^{\top }{\mathbb {E}}[{\varvec{W}}(t)\dot{{\varvec{W}}}(t)^{\top }]\varTheta (t)-\varTheta (t)^{\top }{\mathbb {E}}[{\varvec{W}}(t)\dot{{\varvec{W}}}(t)^{{T}}]\varTheta (t)=0, \end{aligned}$$

where in the penultimate equality we used ${\mathbb {E}}[\dot{{\varvec{W}}}(t){\varvec{W}}(t)^{\top }]+{\mathbb {E}}[{\varvec{W}}(t)\dot{{\varvec{W}}}(t)^{\top }]=0$ ; $t\mapsto \varTheta (t)^{\top }\varTheta (t)$ is absolutely continuous; and $\varTheta (0)^{\top }\varTheta (0)=I$. With this solution $\varTheta (t)\in O(S)$ of (2.11), let

2.13 $$\begin{aligned} {\varvec{U}}(t):=\varTheta (t)^{\top }{\varvec{V}}(t),\ \text {and }\ {\varvec{Y}}(t):=\varTheta (t)^{\top }{\varvec{W}}(t). \end{aligned}$$

We claim that $({\varvec{U}}(t),{\varvec{Y}}(t))$ is a Dual DO solution. First, we note that ${\varvec{U}}$ is linearly independent, and that ${\varvec{Y}}$ is orthonormal and satisfies the gauge condition. Indeed, we have $\mathrm {det}(\langle {\varvec{U}}(t),{\varvec{U}}(t)^{\top }\rangle )\ne 0$, ${\mathbb {E}}[{\varvec{Y}}(t){\varvec{Y}}(t)^{\top }]=I$, and

$$\begin{aligned} {\mathbb {E}}[{\varvec{Y}}(t)\dot{{\varvec{Y}}}(t)^{\top }]&=\varTheta (t)^{\top }{\mathbb {E}}[{\varvec{W}}(t){\varvec{W}}(t)^{\top }]{\dot{\varTheta }}(t)+\varTheta (t)^{\top }{\mathbb {E}}[{\varvec{W}}(t)\dot{{\varvec{W}}}(t)^{\top }]\varTheta (t)\\&=\varTheta (t)^{\top }\big ({\dot{\varTheta }}(t)+{\mathbb {E}}[{\varvec{W}}(t)\dot{{\varvec{W}}}(t)^{\top }]\varTheta (t)\big )=0, \end{aligned}$$

where in the penultimate line we used ${\mathbb {E}}[{\varvec{W}}(t){\varvec{W}}(t)^{\top }]=I$. Then, noting that ${\varvec{U}}(t)^{\top }{\varvec{Y}}(t)={\varvec{V}}(t)^{\top }{\varvec{W}}(t)=u_{S}(t)$ satisfies the original equation (2.4), from the derivation of the Dual DO equation (2.6) (see [17], also [16, 20]) we conclude that $({\varvec{U}}(t),{\varvec{Y}}(t))$ satisfies (2.6). From (2.13), we see that on the compact interval [0, *T*] the functions $t\mapsto {\varvec{U}}(t)\in [{\mathcal {H}}]^S$ and $t\mapsto {\varvec{Y}}(t)\in [L^2(\varOmega )]^S$ are absolutely continuous, and thus $({\varvec{U}}(t),{\varvec{Y}}(t))$ is a strong Dual DO solution.

The uniqueness of the Dual DO solution follows from Corollary 2.4 and the uniqueness of the solution of the Eq. (2.11). $\square$

We are ready to state the following equivalence of the original problem (2.4) and the Dual DO formulation (Definitions 2.3–2.4).

#### Proposition 2.6

Suppose that a strong (resp. classical) DLR solution $u_{S}(t)\in M_{S}$, $t\in [0,T]$ uniquely exists. Then, given the decomposition $({\varvec{U}}_{0},{\varvec{Y}}_{0})\in [{\mathcal {H}}]^{S}\times [L^{2}(\varOmega )]^{S}$ of the initial condition ${u_{0S}}={\varvec{U}}_{0}^{\top }{\varvec{Y}}_{0}\in M_{S}$, the Dual DO solution with the initial condition $({\varvec{U}}_{0},{\varvec{Y}}_{0})$ uniquely exists in the strong sense (resp. the classical sense). Conversely, the unique existence of the Dual DO solution in the strong sense (resp. the classical sense) implies the unique existence of the DLR solution.

#### Proof

The first direction is a direct consequence of the previous lemma for strong solutions. Suppose that the Dual DO solution $({\varvec{U}}(t),{\varvec{Y}}(t))_{t\in [0,T]}$ uniquely exists in the strong sense. Then, from the derivation of the Dual DO equation (2.6), $t\mapsto {\varvec{U}}^{\!\top }\!\!\,(t){\varvec{Y}}(t)\in M_{S}$ is a solution of the original equation (2.4).

Now, we show the uniqueness. Suppose that $t\mapsto {\hat{u}}_{S}(t)\ne {\varvec{U}}^{\top }(t){\varvec{Y}}(t)$ is a DLR solution. From Lemma 2.5, there exists a unique Dual DO solution $(\hat{{\varvec{U}}},\hat{{\varvec{Y}}})$ associated with ${\hat{u}}_{S}$ and the decomposition ${\hat{u}}_{S}(0)={\varvec{U}}_0^{\top }{\varvec{Y}}_0$, i.e. $(\hat{{\varvec{U}}}(t),\hat{{\varvec{Y}}}(t))$ is a solution of the Dual DO equation (2.6). But from the assumption we must have $(\hat{{\varvec{U}}}(t),\hat{{\varvec{Y}}}(t))=({\varvec{U}}(t),{\varvec{Y}}(t))$, $t\in [0,T]$, and therefore $\hat{{\varvec{U}}}(t)^{\top }\hat{{\varvec{Y}}}(t)={\hat{u}}_{S}(t)={\varvec{U}}(t)^{\top }{\varvec{Y}}(t)=u_{S}(t)$, a contradiction. The argument for the classical solution is analogous. $\square$

### Assumptions

In view of Proposition 2.6, we establish the unique existence of the Dual DO solution. We work under the following assumptions. Assumptions 1 and 2 will be used for the existence in the strong sense, and in addition, Assumption 3 will be used for the classical sense. Further, the stability Assumptions 4 and 5 will be used to establish the extendability of the strong solution, and respectively the classical solution, to the maximal time interval.

#### Assumption 1

$\varLambda :D_{{\mathcal {H}}}(\varLambda )\subset {\mathcal {H}}\rightarrow {\mathcal {H}}$ is a closed linear operator that is densely defined in ${\mathcal {H}}$. Furthermore, $\varLambda$ is the infinitesimal generator of the $C_{0}$ semigroup $\mathrm {e}^{t\varLambda }$ satisfying $\Vert \mathrm {e}^{t\varLambda }\Vert _{{\mathcal {H}}\rightarrow {\mathcal {H}}}\le K_{\varLambda }\mathrm {e}^{-\lambda t}$ for $t\ge 0$, with constants $K_{\varLambda }\ge 1$ and $\lambda \ge 0$.

#### Assumption 2

The mapping $F:L^{2}(\varOmega ;{\mathcal {H}})\rightarrow L^{2}(\varOmega ;{\mathcal {H}})$ is locally Lipschitz continuous on $L^{2}(\varOmega ;{\mathcal {H}})$ in the following sense: for every $r>0$ and every $v_{0}\in L^{2}(\varOmega ;{\mathcal {H}})$ such that $\Vert v_{0}\Vert _{L^{2}(\varOmega ;{\mathcal {H}})}\le q$, there exists a constant $C_{q,r}>0$ such that

$$\begin{aligned} \Vert F(w)-F(w')\Vert _{L^{2}(\varOmega ;{\mathcal {H}})}\le C_{q,r}\Vert w-w'\Vert _{L^{2}(\varOmega ;{\mathcal {H}})} \end{aligned}$$

holds for all $w,w'\in L^{2}(\varOmega ;{\mathcal {H}})$ with $\Vert w-v_{0}\Vert _{L^{2}(\varOmega ;{\mathcal {H}})}\le r$, $\Vert w'-v_{0}\Vert _{L^{2}(\varOmega ;{\mathcal {H}})}\le r$. Furthermore, we assume $\Vert F(v_{0})\Vert _{L^{2}(\varOmega ;{\mathcal {H}})}<C'{}_{q}<\infty$.

In the above assumption, note that given the first condition, the second condition is implied by $\Vert F(a)\Vert _{L^{2}(\varOmega ;{\mathcal {H}})}<\infty$ for a point $a\in L^{2}(\varOmega ;{\mathcal {H}})$.

To establish the existence of the Dual DO solution in the classical sense, we use the following further regularity of *F*.

#### Assumption 3

In addition to Assumption 2, assume that for every $r>0$ and every $v_{0}\in L^{2}(\varOmega ;{\mathcal {H}})$ with $\varLambda v_{0}\in L^{2}(\varOmega ;{\mathcal {H}})$ such that $\Vert \varLambda v_{0}\Vert _{L^{2}(\varOmega ;{\mathcal {H}})}\le q$, there exists a constant $C_{q,r}>0$ such that

$$\begin{aligned} \Vert \varLambda (F(w)-F(w'))\Vert _{L^{2}(\varOmega ;{\mathcal {H}})}\le C{}_{q,r}\Vert \varLambda (w-w')\Vert _{L^{2}(\varOmega ;{\mathcal {H}})} \end{aligned}$$

holds for any $w,w'\in L^{2}(\varOmega ;{\mathcal {H}})$ satisfying $\varLambda w,\varLambda w'\in L^{2}(\varOmega ;{\mathcal {H}})$ with $\Vert \varLambda (w-v_{0})\Vert _{L^{2}(\varOmega ;{\mathcal {H}})}\le r,\Vert \varLambda (w'-v_{0})\Vert _{L^{2}(\varOmega ;{\mathcal {H}})}\le r$. Further, assume $\Vert \varLambda F(v_{0})\Vert _{L^{2}(\varOmega ;{\mathcal {H}})}<C'_{q}<\infty$.

Since $\varLambda$ is closed, $D_{{\mathcal {H}}}(\varLambda )$ admits a Hilbert space structure with respect to the graph inner product ${\langle \cdot ,\cdot \rangle +\langle \varLambda \cdot ,\varLambda \cdot \rangle }$, which we denote ${\mathcal {V}}$. Then, Assumptions 2–3 imply that for a constant ${\tilde{C}}_{q,r}>0$ we have

$$\begin{aligned} \Vert F(w)-F(w')\Vert _{L^{2}(\varOmega ;{\mathcal {V}})}\le {\tilde{C}}_{q,r}\Vert w-w'\Vert _{L^{2}(\varOmega ;{\mathcal {V}})} \end{aligned}$$

for any $w,w'\in {\mathcal {V}}$ satisfying $\Vert w-v_{0}\Vert _{L^{2}(\varOmega ;{\mathcal {V}})}\le r,\Vert w'-v_{0}\Vert _{L^{2}(\varOmega ;{\mathcal {V}})}\le r$, and moreover, $\Vert F(v_{0})\Vert _{L^{2}(\varOmega ;{\mathcal {V}})}<{\tilde{C}}'{}_{q}<\infty$.

The following uniform stability condition will be used to establish the existence of a strong Dual DO solution in the maximal interval . Here, uniform means that the constant $C_{\varLambda ,F}$ below is independent of bounds of *v*.

#### Assumption 4

The pair $(\varLambda ,F)$ satisfies the following: for every $v\in L^{2}(\varOmega ;{\mathcal {H}})$ such that $\varLambda v\in L^{2}(\varOmega ;{\mathcal {H}})$ we have

$$\begin{aligned} {\mathbb {E}}[\langle \varLambda (v)+F(v),v\rangle ]\le C_{\varLambda ,F}(1+\Vert v\Vert _{L^{2}(\varOmega ;{\mathcal {H}})}^{2}). \end{aligned}$$

For example, this condition holds when $\varLambda$ satisfies $\langle \varLambda x,x\rangle \le 0$ for $x\in D_{{\mathcal {H}}}(\varLambda )$ and *F* satisfies the uniform linear growth condition $\Vert F(v)\Vert _{L^{2}(\varOmega ;{\mathcal {H}})}\le C'_{F}(1+\Vert v\Vert _{{L^{2}(\varOmega ;{\mathcal {H}})}})$ for some $C'_{F}>0$.

To establish the existence of the classical Dual DO solution in the maximal interval, we use the following stronger uniform stability condition, where we again note that the constant is independent of bounds of *v*.

#### Assumption 5

For every $v\in L^{2}(\varOmega ;{\mathcal {H}})$ such that $\varLambda v\in L^{2}(\varOmega ;{\mathcal {H}})$ we have

$$\begin{aligned} \Vert \varLambda F(v)\Vert _{L^{2}(\varOmega ;{\mathcal {H}})}\le C_{F}(1+\Vert \varLambda v\Vert _{L^{2}(\varOmega ;{\mathcal {H}})}),\quad \text {where }C_{F}>0\ \text {is independent of }v. \end{aligned}$$

The following examples satisfy the above assumptions.

#### Example 2.1

For a bounded domain $D\subset {\mathbb {R}}^{d}$, let ${\mathcal {H}}=L^{2}(D)$. Further, let ${\tilde{\varLambda }}$ be a second order uniformly elliptic differential operator with zero Dirichlet boundary condition. For the non-linear term, let $a,b\in L^{\infty }(\varOmega ;L^{\infty }(D))$, $c\in L^{2}(\varOmega ;L^{2}(D))$, and let $f:{\mathbb {R}}\rightarrow {\mathbb {R}}$ be a differentiable function such that $\sup _{s\in {\mathbb {R}}}|f'(s)|<\infty$. Consider the following multiplicative and additive noise:

$$\begin{aligned} {\tilde{F}}(v):=a\cdot f(v\cdot b)+c,\ \text {for }v\in L^{2}(\varOmega ;L^{2}(D)), \end{aligned}$$

where $\cdot$ denotes the point-wise multiplication. Then, the pair $({\tilde{\varLambda }},{\tilde{F}})$ satisfies Assumptions 1, 2, and 4.

#### Example 2.2

Let $f(x)=x$. With $a\in \!\!\; L^{\infty }(\varOmega ;W^{\infty ,2}(D))$ and $c\in \!\!\; L^{2}(\varOmega ;L^{2}(D))$, let

$$\begin{aligned} \tilde{{\tilde{F}}}(v):=a\cdot v+c,\ \text {for }v\in L^{2}(\varOmega ;L^{2}(D)). \end{aligned}$$

Then, the pair $({\tilde{\varLambda }},\tilde{{\tilde{F}}})$ satisfies Assumptions 1–5.

### On the choice of the dual DO formulation

To establish uniqueness and existence of the DLR approximation we work with the Dual DO formulation (2.6). We have chosen this formulation with care. This section provides a discussion on choosing a good formulation.

The DLR approach to the stochastic dynamical system such as (1.1) was first introduced by Sapsis and Lermusiaux [20]. The formulation they introduced is called the Dynamically Orthogonal (DO) formulation: they imposed the orthogonality of the spatial basis. Musharbash et al. [16] pointed out that the DO approximation can be related to the MCTDH method, by considering the so-called dynamically double orthogonal (DDO) formulation: yet another equivalent formulation of the DLR approach. Through this relation of the DDO approximation to the MCTDH method, Musharbash et al. further developed an error estimate of the DO method. The error analysis obtained by Musharbash et al. was partially built upon results regarding the MCTDH method.

A reasonable strategy to establish the existence of the DLR approximation would thus be to establish the existence of the DDO approximation. Namely, following the argument of Koch and Lubich [13], it is tempting to apply the gauge condition defined by the differential operator $\varLambda$ to the DDO formulation. It turns out that this approach does not work, since the aforementioned gauge condition turns out to be vacuous unless $\varLambda$ is skew-symmetric, as we illustrate hereafter.

In the DDO formulation, we seek an approximant of the form

$$\begin{aligned} u_{S}(t)=\tilde{{\varvec{U}}}^{\top }(t)A(t){\varvec{Y}}(t), \end{aligned}$$

where $\tilde{{\varvec{U}}}(t)=(U_{1}(t),\dots ,U_{S}(t))^{\top }$, and ${\varvec{Y}}(t)=(Y_{1}(t),\dots ,Y_{S}(t))^{\top }$ are orthonormal in ${\mathcal {H}}$, and in $L^{2}(\varOmega )$ respectively; and $A(t)\in {\mathbb {R}}^{S\times S}$ is a full-rank matrix. The triplet $(\tilde{{\varvec{U}}},A,{\varvec{Y}})$ is given as a solution of the set of equations:

2.14 $$\begin{aligned} \frac{\mathrm {d}}{\mathrm {d}t}A&={\mathbb {E}}\big [\big \langle \varLambda (u_{S})+F(u_{S}),\tilde{{\varvec{U}}}\big \rangle {\varvec{Y}}^{\top }\big ],\nonumber \\ A^{\top }\frac{\mathrm {d}\tilde{{\varvec{U}}}}{\mathrm {d}t}&=(I-P_{\tilde{{\varvec{U}}}})A^{\top }\varLambda (\tilde{{\varvec{U}}})+(I-P_{\tilde{{\varvec{U}}}}){\mathbb {E}}\big [{\varvec{Y}}\big (F(u_{S})\big )\big ],\\ A\frac{\partial {\varvec{Y}}}{\partial t}&=(I-P_{{\varvec{Y}}})\big \langle \varLambda (\tilde{{\varvec{U}}}^{\top })A{\varvec{Y}}+F(u_{S}),\tilde{{\varvec{U}}}\big \rangle ,\nonumber \end{aligned}$$

where $P_{\tilde{{\varvec{U}}}}:{\mathcal {H}}\rightarrow \mathrm {span}\{{\tilde{U}}_{j}:j=1,\dotsc ,S\}$ is the ${\mathcal {H}}$-orthogonal projection onto $\mathrm {span}\{{\tilde{U}}_{j}:j=1,\dotsc S\}$, and $P_{{\varvec{Y}}}:L^{2}(\varOmega )\rightarrow \mathrm {span}\{Y_{j}:j=1,\dotsc ,S\}$ is the $L^{2}(\varOmega )$-orthogonal projection onto $\mathrm {span}\{Y_{j}:j=1,\dotsc S\}$. These equations are derived using the orthonormality assumption on $(\tilde{{\varvec{U}}},{\varvec{Y}})$ together with the gauge conditions

2.15 $$\begin{aligned} \Big \langle \frac{\partial }{\partial t}\tilde{{\varvec{U}}},\tilde{{\varvec{U}}}^{\top }\Big \rangle =0\text { and }{\mathbb {E}}\Big [\Big (\frac{\partial }{\partial t}{\varvec{Y}}\Big ){\varvec{Y}}^{\top }\Big ]=0, \end{aligned}$$

see [16, (3.14)–(3.17)].

We note that in the Eq. (2.14) for $\tilde{{\varvec{U}}}$ we have the composition of the unbounded operator $\varLambda$ and the projection operator $P_{\tilde{{\varvec{U}}}}$, where we note that the map $\tilde{{\varvec{U}}}\mapsto P_{\tilde{{\varvec{U}}}}$ is non-linear. Koch and Lubich [13] had a similar situation in the MCTDH setting. As outlined above, they got away with this problem by considering a different gauge condition. We will explain below an analogous strategy and why it does not work in our setting.

First, from the orthonormality condition on $\tilde{{\varvec{U}}}$ it is necessary to have $\frac{\mathrm {d}}{\mathrm {d}t}\langle \tilde{{\varvec{U}}},\tilde{{\varvec{U}}}^{\top }\rangle =0$. The above gauge condition (2.15) on $\tilde{{\varvec{U}}}$ is sufficient for this to hold. But since

$$\begin{aligned} \frac{\mathrm {d}}{\mathrm {d}t}\langle \tilde{{\varvec{U}}},\tilde{{\varvec{U}}}^{\top }\rangle =\langle \frac{\partial }{\partial t}\tilde{{\varvec{U}}},\tilde{{\varvec{U}}}^{\top }\rangle +\langle \tilde{{\varvec{U}}},\frac{\partial }{\partial t}\tilde{{\varvec{U}}}^{\top }\rangle , \end{aligned}$$

the solution $\tilde{{\varvec{U}}}$ stays orthonormal if and only if we impose the gauge condition $\langle \frac{\partial }{\partial t}\tilde{{\varvec{U}}},\tilde{{\varvec{U}}}^{\top }\rangle =-\langle \tilde{{\varvec{U}}},\frac{\partial }{\partial t}\tilde{{\varvec{U}}}^{\top }\rangle$. Koch and Lubich [13] noted this, and to establish an existence result they considered a suitable gauge condition, which enabled them to take the differential operator out of the projection. The gauge condition that is formally analogous to [13] may be given as $\langle \frac{\partial }{\partial t}\tilde{{\varvec{U}}},\tilde{{\varvec{U}}}^{\top }\rangle =\langle \varLambda \tilde{{\varvec{U}}},\tilde{{\varvec{U}}}^{\top }\rangle$, for $\varLambda$ not necessarily skew-symmetric. One can check that this condition formally allows us to take the operator $\varLambda$ out of the projection $P_{\tilde{{\varvec{U}}}}$, but for example when $\varLambda$ is self-adjoint, the solution $\tilde{{\varvec{U}}}$ will not stay orthonormal. This is not acceptable, since we use the orthonormality to derive the Eq. (2.14), and thus we necessarily have to consider a different gauge condition or a different formulation.

## Parameter equation

This section introduces the parameter equation, for which we establish the unique existence of the solution later in Sect. 4. Consider the direct sum of the Hilbert spaces ${{\mathcal {X}}:=}[{\mathcal {H}}]^{S}\oplus [L^{2}(\varOmega )]^{S}$ equipped with the inner product $\langle (\hat{{\varvec{U}}},\hat{{\varvec{Y}}}),(\hat{{\varvec{V}}},\hat{{\varvec{W}}})\rangle _{{\mathcal {X}}}:=\langle \hat{{\varvec{U}}},\hat{{\varvec{V}}}\rangle _{[{\mathcal {H}}]^{S}}+\langle \hat{{\varvec{Y}}},\hat{{\varvec{W}}}\rangle _{[L^{2}(\varOmega )]^{S}}$. In what follows, we redefine the operator $\varLambda$ as $\varLambda :D_{{\mathcal {H}}}(\varLambda )\subset [{\mathcal {H}}]^{S}\rightarrow [{\mathcal {H}}]^{S}$, ${\varvec{U}}\mapsto (\varLambda U_{1},\dots ,\varLambda U_{S})=:\varLambda {\varvec{U}}$ for ${\varvec{U}}\in D_{{\mathcal {H}}}(\varLambda )\subset [{\mathcal {H}}]^{S}$. We define the linear operator $A:{\mathcal {X}}\rightarrow {\mathcal {X}}$ by

$$\begin{aligned} A(\hat{{\varvec{U}}},\hat{{\varvec{Y}}})=(\varLambda \hat{{\varvec{U}}},0)\quad \text {for }(\hat{{\varvec{U}}},\hat{{\varvec{Y}}})\in {\mathcal {X}}, \end{aligned}$$

with $D(A)=D_{{\mathcal {H}}}(\varLambda )\oplus [L^{2}(\varOmega )]^{S}$. Further, we define $G:D(G)\subset {\mathcal {X}}\rightarrow {\mathcal {X}}$ by

3.1 $$\begin{aligned} G(\hat{{\varvec{U}}},\hat{{\varvec{Y}}})&:=\big ([G_{1}(\hat{{\varvec{Y}}})](\hat{{\varvec{U}}}),[G_{2}(\hat{{\varvec{U}}})](\hat{{\varvec{Y}}})\big )\nonumber \\&:=\big ({\mathbb {E}}\big [F(\hat{{\varvec{U}}}^{\top }\hat{{\varvec{Y}}})\hat{{\varvec{Y}}}\big ],(I-P_{\hat{{\varvec{Y}}}})\big (\langle F(\hat{{\varvec{U}}}^{\top }\hat{{\varvec{Y}}}),Z_{\hat{{\varvec{U}}}}^{-1}\hat{{\varvec{U}}}\rangle \big )\big ), \end{aligned}$$

where $$D(G):=\{(\hat{{\varvec{U}}},\hat{{\varvec{Y}}})\in {\mathcal {X}}\mid Z_{\hat{{\varvec{U}}}}^{-1}\text { exists}\}$$.

Then, the Dual DO solution, if it exists, satisfies the following Cauchy problem for a semi-linear abstract evolution equation in ${\mathcal {X}}$:

3.2 $$\begin{aligned} \left\{ \begin{array}{l} \frac{\mathrm {d}}{\mathrm {d}t}({\varvec{U}},{\varvec{Y}}) =A({\varvec{U}},{\varvec{Y}})+G({\varvec{U}},{\varvec{Y}})\quad \text {for}\ t>0,\\ ({\varvec{U}}(0),{\varvec{Y}}(0)) =({\varvec{U}}_{0},{\varvec{Y}}_{\!0}), \end{array}\right. \end{aligned}$$

where the initial condition $({\varvec{U}}_{0},{\varvec{Y}}_{\!0})\in {\mathcal {X}}$ satisfies suitable assumptions detailed below. Conversely, later in Sect. 4 we will see that the strong solution of this Cauchy problem is a Dual DO solution, and that it gives a DLR solution. We first establish the unique existence of the mild solution of (3.2):

$$\begin{aligned} {\varvec{U}}(t)&=e^{t\varLambda }{\varvec{U}}(0)+\int _{0}^{t}e^{(t-\tau )\varLambda }\big [G_{1}\big ({\varvec{Y}}(\tau )\big )\big ]\big ({\varvec{U}}(\tau )\big )\mathrm {d}\tau ,\\ {\varvec{Y}}(t)&={\varvec{Y}}(0)+\int _{0}^{t}\big [G_{2}\big ({\varvec{U}}(\tau )\big )\big ]\big ({\varvec{Y}}(\tau )\big )\mathrm {d}\tau . \end{aligned}$$

We will use the following result, which is a variation of a standard local existence and uniqueness theorem for mild solutions, e.g. see [18, Theorem 6.1.4] or [21, Theorem 46.1], adapted to our setting.

### Proposition 3.1

Suppose that the operator $A:D(A)\subset {\mathcal {X}}\rightarrow {\mathcal {X}}$ generates a $C_{0}$ semigroup $\mathrm {e}^{tA}$, $t\ge 0$ on ${\mathcal {X}}$. Suppose further that the mapping $G:{\mathcal {X}}\rightarrow {\mathcal {X}}$ is locally Lipschitz continuous on ${\mathcal {X}}$ in the following sense: for an element $(\hat{{\varvec{U}}},\hat{{\varvec{Y}}})\in {\mathcal {X}}$ with $\alpha \ge \Vert \hat{{\varvec{U}}}\Vert _{[{\mathcal {H}}]^{S}}$ and $\beta \ge \Vert \hat{{\varvec{Y}}}\Vert _{[L^{2}(\varOmega )]^{S}}$, there exists $r=r(\hat{{\varvec{U}}},\hat{{\varvec{Y}}})>0$ and $C_{\alpha ,\beta }>0$ such that

$$\begin{aligned} \Vert G({\varvec{V}},{\varvec{W}})-G({\varvec{V}}',{\varvec{W}}')\Vert _{{\mathcal {X}}}\le C_{\alpha ,\beta }\Vert ({\varvec{V}},{\varvec{W}})-({\varvec{V}}',{\varvec{W}}')\Vert _{{\mathcal {X}}} \end{aligned}$$

holds for all $({\varvec{V}},{\varvec{W}}),({\varvec{V}}',{\varvec{W}}')\in {\mathcal {X}}$ with $\Vert {\varvec{W}}-\hat{{\varvec{Y}}}\Vert _{[L^{2}(\varOmega )]^{S}}\le r$ and $\Vert {\varvec{W}}'-\hat{{\varvec{Y}}}\Vert _{[L^{2}(\varOmega )]^{S}}\le r$; $\Vert {\varvec{V}}-\hat{{\varvec{U}}}\Vert _{[{\mathcal {H}}]^{S}}\le r$ and $\Vert {\varvec{V}}'-\hat{{\varvec{U}}}\Vert _{[{\mathcal {H}}]^{S}}\le r$. Further, suppose that for some $C'_{\alpha ,\beta }>0$ we have $\Vert G(\hat{{\varvec{U}}},\hat{{\varvec{Y}}})\Vert _{{\mathcal {X}}}\le C'_{\alpha ,\beta }$. Then, the problem (3.2) starting at $t_0\ge 0$ with the initial condition $(\hat{{\varvec{U}}},\hat{{\varvec{Y}}})\in {{\mathcal {X}}}$:

$$\begin{aligned} \left\{ \begin{array}{l} \frac{\mathrm {d}}{\mathrm {d}t}({\varvec{U}},{\varvec{Y}}) =A({\varvec{U}},{\varvec{Y}})+G({\varvec{U}},{\varvec{Y}})\quad \text {for}\ t>t_0,\\ ({\varvec{U}}(t_0),{\varvec{Y}}(t_0)) =(\hat{{\varvec{U}}},\hat{{\varvec{Y}}}), \end{array}\right. \end{aligned}$$

has a unique mild solution on an interval of length $\delta \in (0,1]$, where $\delta$ depends on $\alpha$, $\beta$, $\sup _{s{\in [t_0,t_0+1]}}\Vert \mathrm {e}^{sA}\Vert$, and $r=r(\hat{{\varvec{U}}},\hat{{\varvec{Y}}})$.

To invoke this proposition, we start with checking that the operator *A* defined above generates a $C_{0}$ semigroup.

### Proposition 3.2

Let Assumption 1 hold. Then, $A:D(A)\subset {\mathcal {X}}\rightarrow {\mathcal {X}}$ generates a $C_{0}$ semigroup $\mathrm {e}^{tA}$, $t\ge 0$ on ${\mathcal {X}}$ with the bound $\Vert \mathrm {e}^{tA}\Vert _{{\mathcal {X}}\rightarrow {\mathcal {X}}}\le K_{\varLambda }$.

### Proof

We note that $D(A)=D_{{\mathcal {H}}}(\varLambda )\oplus [L^{2}(\varOmega )]^{S}$ is dense in ${\mathcal {X}}$. Further, the closedness of $\varLambda :D_{{\mathcal {H}}}(\varLambda )\subset [{\mathcal {H}}]^{S}\rightarrow [{\mathcal {H}}]^{S}$ implies that $A:D(A)\subset {\mathcal {X}}\rightarrow {\mathcal {X}}$ is closed.

We will invoke the Hille–Yosida theorem, see for example [18, Theorem 1.5.2]. From Assumption 1, every $\mu >0$ is in the resolvent set of $\varLambda$. Thus, $(\mu I-\varLambda )^{-1}:[{\mathcal {H}}]^{S}\rightarrow [{\mathcal {H}}]^{S}$ as well as $(\mu I-0)^{-1}=\frac{1}{\mu }:[L^{2}(\varOmega )]^{S}\rightarrow [L^{2}(\varOmega )]^{S}$ are well-defined, and so is $(\mu I-A)^{-1}$. For any $(\hat{{\varvec{U}}},\hat{{\varvec{Y}}})\in {{\mathcal {X}}}$, ${n\in {\mathbb {N}}}$ we have

$$\begin{aligned} \Vert (\mu I-A)^{-{n}}(\hat{{\varvec{U}}},\hat{{\varvec{Y}}})\Vert _{{\mathcal {X}}}^{2}=\Vert (\mu I-\varLambda )^{-{n}}\hat{{\varvec{U}}}\Vert _{[{\mathcal {H}}]^{S}}^{2}+\frac{1}{\mu ^{2{n}}}\Vert \hat{{\varvec{Y}}}\Vert _{[L^{2}(\varOmega )]^{S}}^{2}, \end{aligned}$$

but Assumption 1 implies ${\Vert (\mu I-\varLambda )^{-n}\Vert _{[{\mathcal {H}}]^{S}}\le K_{\varLambda }/\mu ^{n}}$, and thus we obtain

$$\begin{aligned} \Vert (\mu I-A)^{-{n}}(\hat{{\varvec{U}}},\hat{{\varvec{Y}}})\Vert _{{\mathcal {X}}}^{2} \le \frac{{K_{\varLambda }^{2}}}{\mu ^{2{n}}}\Vert (\hat{{\varvec{U}}},\hat{{\varvec{Y}}})\Vert _{{\mathcal {X}}}^{2}. \end{aligned}$$

In view of the Hille–Yosida theorem the statement now follows. $\square$

Furthermore, we establish a Lipschitz continuity of the non-linear term *G*. We start with the Lipschitz continuity of the projection operator.

### Lemma 3.3

For $\hat{{\varvec{Y}}}=({\hat{Y}}_{1},\dots ,{\hat{Y}}_{S})^{\top }\in [L^{2}(\varOmega )]^{S}$, suppose that the smallest eigenvalue $\sigma _{\hat{{\varvec{Y}}}}$ of the Gram matrix ${\mathbb {E}}[\hat{{\varvec{Y}}}\hat{{\varvec{Y}}}^{\top }]$ is non-zero. Further, let $\kappa \in (0,{\overline{\kappa }})$ be given, where with $\beta \ge \Vert \hat{{\varvec{Y}}}\Vert _{[L^{2}(\varOmega )]^{S}}$, we let

3.3 $$\begin{aligned} {\overline{\kappa }} :={\overline{\kappa }}(\sigma _{\hat{{\varvec{Y}}}},\beta ) :=\frac{1}{2}\big (-\beta +\sqrt{\beta ^{2}+{\sigma _{\hat{{\varvec{Y}}}}}}\big ). \end{aligned}$$

Then, we have

3.4 $$\begin{aligned} \Vert (I-P_{\hat{{\varvec{W}}}'})P_{\hat{{\varvec{W}}}}\Vert _{[L^{2}(\varOmega )]^{S}\rightarrow [L^{2}(\varOmega )]^{S}}\le C_{\kappa ,\beta ,\sigma _{\hat{{\varvec{Y}}}}}\Vert (\hat{{\varvec{W}}}-\hat{{\varvec{W}}}')\Vert _{[L^{2}(\varOmega )]^{S}}<1 \end{aligned}$$

for any $\hat{{\varvec{W}}},\hat{{\varvec{W}}}'\in [L^{2}(\varOmega )]^{S}$ with $\Vert \hat{{\varvec{W}}}-\hat{{\varvec{Y}}}\Vert _{[L^{2}(\varOmega )]^{S}}\le \kappa$, $\Vert \hat{{\varvec{W}}}'-\hat{{\varvec{Y}}}\Vert _{[L^{2}(\varOmega )]^{S}}\le \kappa$, where $C_{\kappa ,\beta ,\sigma _{\hat{{\varvec{Y}}}}} :={2({\kappa +\beta })/\sigma _{\hat{{\varvec{Y}}}}}$.

### Proof

We first show that the smallest eigenvalue $\sigma _{\hat{{\varvec{W}}}}$ of the Gramian ${\mathbb {E}}[\hat{{\varvec{W}}}\hat{{\varvec{W}}}^{\top }]$ is positive, and thus in particular ${\mathbb {E}}[\hat{{\varvec{W}}}\hat{{\varvec{W}}}^{\top }]$ is non-singular. We have

$$\begin{aligned} \frac{-\beta +\sqrt{\beta ^{2}+\frac{\sigma _{\hat{{\varvec{Y}}}}}{2}}}{ \frac{1}{2}(-\beta +\sqrt{\beta ^{2}+{\sigma _{\hat{{\varvec{Y}}}}}})} \ge \frac{2}{\sigma _{\hat{{\varvec{Y}}}}}\Big (\beta ^2+\frac{\sigma _{\hat{{\varvec{Y}}}}}{2}-\beta ^2\Big )= 1, \end{aligned}$$

and thus the assumption on $\kappa$ implies $\kappa ^{2}+2\kappa \beta <\frac{\sigma _{\hat{{\varvec{Y}}}}}{2}$. On the other hand, we have

$$\begin{aligned} \Vert {\mathbb {E}}[\hat{{\varvec{W}}}\hat{{\varvec{W}}}^{\top }]-{\mathbb {E}}[\hat{{\varvec{Y}}}\hat{{\varvec{Y}}}^{\top }]\Vert _{\mathrm {F}}\le \Vert \hat{{\varvec{W}}}\Vert _{[L^{2}(\varOmega )]^{S}}\kappa +\kappa \beta \le (\kappa +\beta )\kappa +\kappa \beta . \end{aligned}$$

Therefore, we obtain $\Vert {\mathbb {E}}[\hat{{\varvec{W}}}\hat{{\varvec{W}}}^{\top }]-{\mathbb {E}}[\hat{{\varvec{Y}}}\hat{{\varvec{Y}}}^{\top }]\Vert _{\mathrm {F}}<\frac{\sigma _{\hat{{\varvec{Y}}}}}{2}$. From the inequality $|\sigma _{\hat{{\varvec{Y}}}}-\sigma _{\hat{{\varvec{W}}}}|\le \Vert {\mathbb {E}}[\hat{{\varvec{Y}}}\hat{{\varvec{Y}}}^{\top }]-{\mathbb {E}}[\hat{{\varvec{W}}}\hat{{\varvec{W}}}^{\top }]\Vert _{\mathrm {F}}$, e.g. [8, Corollary 7.3.5], we conclude

3.5 $$\begin{aligned} 0<\frac{\sigma _{\hat{{\varvec{Y}}}}}{2}<\sigma _{\hat{{\varvec{W}}}}. \end{aligned}$$

Next, we note that the identity

$$\begin{aligned} (I-P_{\hat{{\varvec{W}}}'})P_{\hat{{\varvec{W}}}}g=(I-P_{\hat{{\varvec{W}}}'})(\hat{{\varvec{W}}}-\hat{{\varvec{W}}}')^{\top }\big ({\mathbb {E}}[\hat{{\varvec{W}}}\hat{{\varvec{W}}}^{\top }]\big )^{-1}{\mathbb {E}}[\hat{{\varvec{W}}}g] \end{aligned}$$

holds for any $g\in L^{2}(\varOmega )$: indeed, we have $(I-P_{\hat{{\varvec{W}}}'})(\hat{{\varvec{W}}}-\hat{{\varvec{W}}}')^{\top } =(I-P_{\hat{{\varvec{W}}}'})\hat{{\varvec{W}}}^{\top }$, but $\hat{{\varvec{W}}}^{\top }\big ({\mathbb {E}}[\hat{{\varvec{W}}}\hat{{\varvec{W}}}^{\top }]\big )^{-1}{\mathbb {E}}[\hat{{\varvec{W}}}g]=P_{\hat{{\varvec{W}}}}g$. This type of identity was shown by Wedin in the finite dimensional setting, see [22, (4.2)]. In view of this identity, the first inequality in (3.4) can be shown as

3.6 $$\begin{aligned}&\Vert (\hat{{\varvec{W}}}-\hat{{\varvec{W}}}')^{\top } \big ({\mathbb {E}}[\hat{{\varvec{W}}}\hat{{\varvec{W}}}^{\top }]\big )^{-1}{\mathbb {E}}[\hat{{\varvec{W}}}{\varvec{g}}^{\top }]\Vert _{[L^{2}(\varOmega )]^{S}}\nonumber \\&\quad \le \Vert (\hat{{\varvec{W}}}-\hat{{\varvec{W}}}')\Vert _{[L^{2}(\varOmega )]^{S}}\frac{{2}}{\sigma _{\hat{{\varvec{Y}}}}}(\kappa +\beta )\Vert {\varvec{g}}\Vert _{[L^{2}(\varOmega )]^{S}}, \end{aligned}$$

where we used the assumption on $\hat{{\varvec{W}}},\hat{{\varvec{W}}}'$ and (3.5). Finally, we apply the inequality $\Vert (\hat{{\varvec{W}}}-\hat{{\varvec{W}}}')\Vert _{[L^{2}(\varOmega )]^{S}}\le 2\kappa$ to (3.6). Then, noting that the assumption on $\kappa$ implies $\kappa ^{2}+\beta \kappa <\frac{\sigma _{\hat{{\varvec{Y}}}}}{{4}}$, we have $\Vert (\hat{{\varvec{W}}}-\hat{{\varvec{W}}}')\Vert _{[L^{2}(\varOmega )]^{S}} \bigl (\frac{{2}}{\sigma _{\hat{{\varvec{Y}}}}}\bigr )(\kappa +\beta ) \le \bigl (\frac{{4}}{\sigma _{\hat{{\varvec{Y}}}}}\bigr )(\kappa ^{2}+\kappa \beta ) <1$, which completes the proof. $\square$

### Lemma 3.4

Under the assumptions of Lemma 3.3, we have

$$\begin{aligned} \Vert P_{\hat{{\varvec{W}}}}-P_{\hat{{\varvec{W}}}'}\Vert _{[L^{2}(\varOmega )]^{S}\rightarrow [L^{2}(\varOmega )]^{S}} \le C_{\kappa ,\beta ,\sigma _{\hat{{\varvec{Y}}}}}\Vert \hat{{\varvec{W}}}-\hat{{\varvec{W}}}'\Vert _{[L^{2}(\varOmega )]^{S}} \end{aligned}$$

for any $\hat{{\varvec{W}}},\hat{{\varvec{W}}}'\in [L^{2}(\varOmega )]^{S}$ with $\Vert \hat{{\varvec{W}}}-\hat{{\varvec{Y}}}\Vert _{[L^{2}(\varOmega )]^{S}}\le \kappa$, $\Vert \hat{{\varvec{W}}}'-\hat{{\varvec{Y}}}\Vert _{[L^{2}(\varOmega )]^{S}}\le \kappa$, where $\kappa \in (0,{\overline{\kappa }}(\sigma _{\hat{{\varvec{Y}}}},\beta ))$ and $C_{\kappa ,\beta ,\sigma _{\hat{{\varvec{Y}}}}}$ are as in Lemma 3.3.

### Proof

In view of Lemma 3.3, it suffices to show $\Vert P_{\hat{{\varvec{W}}}}-P_{\hat{{\varvec{W}}}'}\Vert _{[L^{2}(\varOmega )]^{S}\rightarrow [L^{2}(\varOmega )]^{S}}=\Vert (I-P_{\hat{{\varvec{W}}}'})P_{\hat{{\varvec{W}}}}\Vert _{[L^{2}(\varOmega )]^{S}\rightarrow [L^{2}(\varOmega )]^{S}}$. We will invoke a perturbation result on pairs of projections, [10, Lemma 221], see also [9, Theorem I.6.34]. In this regard, first we will show the following identity of finite dimensional vector subspaces

3.7 $$\begin{aligned} \mathrm {Im}\bigl (P_{\hat{{\varvec{W}}}'}|_{\mathrm {Im}(P_{\hat{{\varvec{W}}}})}\bigr )\! :=\! P_{\hat{{\varvec{W}}}'}\bigl (P_{\hat{{\varvec{W}}}}([L^{2}(\varOmega )]^{S})\bigr ) \!\,=\!\,P_{\hat{{\varvec{W}}}'}([L^{2}(\varOmega )]^{S}) =:\!\mathrm {Im}(P_{\hat{{\varvec{W}}}'}). \end{aligned}$$

It suffices to show that $\mathrm {Im}(P_{\hat{{\varvec{W}}}'}|_{\mathrm {Im}(P_{\hat{{\varvec{W}}}})})$ cannot be a proper subspace of $\mathrm {Im}(P_{\hat{{\varvec{W}}}'})$. We will verify that the dimension of $\mathrm {Im}(P_{\hat{{\varvec{W}}}'}|_{\mathrm {Im}(P_{\hat{{\varvec{W}}}})})$ and $\mathrm {Im}(P_{\hat{{\varvec{W}}}'})$ are the same. In view of (3.5) in the proof of Lemma 3.3, we have

$$\begin{aligned} \mathrm {dim}(\mathrm {Im}(P_{\hat{{\varvec{W}}}'}))=S=\mathrm {dim}(\mathrm {Im}(P_{\hat{{\varvec{W}}}})). \end{aligned}$$

Therefore, if the linear operator $P_{\hat{{\varvec{W}}}'}|_{\mathrm {Im}(P_{\hat{{\varvec{W}}}})}:\mathrm {Im}(P_{\hat{{\varvec{W}}}})\rightarrow \mathrm {Im}(P_{\hat{{\varvec{W}}}'}|_{\mathrm {Im}(P_{\hat{{\varvec{W}}}})})$ is a vector space isomorphism, then we have $\mathrm {dim}(\mathrm {Im}(P_{\hat{{\varvec{W}}}'}|_{\mathrm {Im}(P_{\hat{{\varvec{W}}}})}))=S$, and thus (3.7) will follow. It suffices to show the injectivity. For any ${\varvec{x}}=P_{\hat{{\varvec{W}}}}{\varvec{x}}\in \mathrm {Im}(P_{\hat{{\varvec{W}}}})$, with $d:=\Vert (I-P_{\hat{{\varvec{W}}}'})P_{\hat{{\varvec{W}}}}\Vert _{[L^{2}(\varOmega )]^{S}\rightarrow [L^{2}(\varOmega )]^{S}}$ we have

$$\begin{aligned} \Vert {\varvec{x}}-P_{\hat{{\varvec{W}}}'}{\varvec{x}}\Vert _{[L^{2}(\varOmega )]^{S}}&=\Vert P_{\hat{{\varvec{W}}}}{\varvec{x}}-P_{\hat{{\varvec{W}}}'}P_{\hat{{\varvec{W}}}}{\varvec{x}}\Vert _{[L^{2}(\varOmega )]^{S}} \le d\Vert {\varvec{x}}\Vert _{[L^{2}(\varOmega )]^{S}}, \end{aligned}$$

where from (3.4) we have $d<1$. Thus, we get $\Vert {\varvec{x}}\Vert _{[L^{2}(\varOmega )]^{S}}{\le }\frac{1}{1-d}\Vert P_{\hat{{\varvec{W}}}'}{\varvec{x}}\Vert _{[L^{2}(\varOmega )]^{S}}$, which shows the injectivity. Hence we have (3.7). Finally, in view of [10, i) Lemma 221], we have

$$\begin{aligned} \Vert P_{\hat{{\varvec{W}}}}-P_{\hat{{\varvec{W}}}'}\Vert _{[L^{2}(\varOmega )]^{S}\rightarrow [L^{2}(\varOmega )]^{S}}=\Vert (I-P_{\hat{{\varvec{W}}}'})P_{\hat{{\varvec{W}}}}\Vert _{[L^{2}(\varOmega )]^{S}\rightarrow [L^{2}(\varOmega )]^{S}}, \end{aligned}$$

and the statement follows from Lemma 3.3. $\square$

Next, we derive a local Lipschitz continuity of the inverse of $Z_{\hat{{\varvec{U}}}}=\langle \hat{{\varvec{U}}},\hat{{\varvec{U}}}^{\top }\rangle$.

### Lemma 3.5

Suppose that $\hat{{\varvec{U}}},\hat{{\varvec{U}}}'\in [{\mathcal {H}}]^{S}$ are linearly independent and that for some ${\tilde{\alpha }}>0$ we have $\max \{\Vert \hat{{\varvec{U}}}\Vert _{[{\mathcal {H}}]^{S}},\Vert \hat{{\varvec{U}}}'\Vert _{[{\mathcal {H}}]^{S}}\}\le {\tilde{\alpha }}$. Then, it holds

$$\begin{aligned} {\Vert Z_{\hat{{\varvec{U}}}}^{-1}-Z_{\hat{{\varvec{U}}}'}^{-1}\Vert _{{2}}\le C_{{\tilde{\alpha }},S}(\Vert Z_{\hat{{\varvec{U}}}}^{-1}\Vert _{{2}}^{2}+\Vert Z_{\hat{{\varvec{U}}'}}^{-1}\Vert _{{2}}^{2})\Vert \hat{{\varvec{U}}}-\hat{{\varvec{U}}}'\Vert _{[{\mathcal {H}}]^{S}}} \end{aligned}$$

with a constant $$C_{{\tilde{\alpha }},S}>0$$.

### Proof

For components ${\hat{U}}_{j}$, ${\hat{U}}_{k}$ of $\hat{{\varvec{U}}}$; and ${\hat{U}}'_{j}$, ${\hat{U}}'_{k}$ of $\hat{{\varvec{U}}}'$, we have

$$\begin{aligned} |\langle {\hat{U}}_{j},{\hat{U}}{}_{k}\rangle -\langle {\hat{U}}'_{j},{\hat{U}}'_{k}\rangle |\le \max \{\Vert {\hat{U}}_{k}\Vert _{{\mathcal {H}}},\Vert {\hat{U}}'_{j}\Vert _{{\mathcal {H}}}\}(\Vert {\hat{U}}{}_{j}-{\hat{U}}'_{j}\Vert _{{\mathcal {H}}}+\Vert {\hat{U}}{}_{k}-{\hat{U}}'_{k}\Vert _{{\mathcal {H}}}), \end{aligned}$$

and thus there exists a constant $C'_{{\tilde{\alpha }},S}$ depending on *S* such that $\Vert Z_{\hat{{\varvec{U}}}}-Z_{\hat{{\varvec{U}}}'}\Vert _{2} \le \Vert Z_{\hat{{\varvec{U}}}}-Z_{\hat{{\varvec{U}}}'}\Vert _{\mathrm {F}}\le C'_{{\tilde{\alpha }},S}\Vert \hat{{\varvec{U}}}-\hat{{\varvec{U}}}'\Vert _{[{\mathcal {H}}]^{S}}$.

Noting that the matrix $Z_{\hat{{\varvec{U}}}}$ is non-singular when $\hat{{\varvec{U}}}$ is linear independent, we recall that the Fréchet derivative of the mapping $\mathbb {{R}}^{S\times S}\ni B\mapsto B^{-1}=:\mathrm {Inv}(B)\in \mathbb {{R}}^{S\times S}$ at *B* acting on $W\in {\mathbb {R}}^{S\times S}$ is given by $D\,\mathrm {Inv}(B)[W]=-B^{-1}WB^{-1}$ (see, e.g. [1, Appendix A.5]). Then, with the notation $\Vert D\,\mathrm {Inv}(Z_{\hat{{\varvec{U}}}})\Vert _{{\mathbb {R}}^{S\times S}\rightarrow {\mathbb {R}}^{S\times S}} :=\max _{W\in {\mathbb {R}}^{S\times S}:\Vert W\Vert _{{2}}=1}\Vert Z_{\hat{{\varvec{U}}}}^{-1}WZ_{\hat{{\varvec{U}}}}^{-1}\Vert _{{2}}$, in view of [3, Corollary 3.2] we have

$$\begin{aligned} \Vert Z_{\hat{{\varvec{U}}}}^{-1} -Z_{\hat{{\varvec{U}}}'}^{-1}\Vert _{{2}}&=\Vert \mathrm {Inv}(Z_{\hat{{\varvec{U}}}})-\mathrm {Inv}(Z_{\hat{{\varvec{U}}}'})\Vert _{{2}}\\&\le \sup \Big \{\Vert D\,\mathrm {Inv}({\tilde{Z}})\Vert _{{\mathbb {R}}^{S\times S}\rightarrow {\mathbb {R}}^{S\times S}}\,\Big |\,{\tilde{Z}}=rZ_{\hat{{\varvec{U}}}}+(1-r)Z_{\hat{{\varvec{U}}}'},\,r\in [0,1]\Big \}\\&\quad \times \Vert Z_{\hat{{\varvec{U}}}}-Z_{\hat{{\varvec{U}}}'}\Vert _{{2}}\\&\le \sup \Big \{\Vert {\tilde{Z}}^{-1}\Vert _{{2}}^{2}\,\Big |\,{\tilde{Z}}=rZ_{\hat{{\varvec{U}}}}+(1-r)Z_{\hat{{\varvec{U}}}'},\,r\in [0,1]\Big \}\Vert Z_{\hat{{\varvec{U}}}}-Z_{\hat{{\varvec{U}}}'}\Vert _{{2}}. \end{aligned}$$

Now, for $r\in [0,1]$ given, since $Z_{\hat{{\varvec{U}}}}^{-1}$ and $Z_{\hat{{\varvec{U}}}'}^{-1}$ are symmetric positive definite, from [15] we have ${\varvec{c}}^\top \big (rZ_{\hat{{\varvec{U}}}}+(1-r)Z_{\hat{{\varvec{U}}}'}\big )^{-1} {\varvec{c}} \le r{\varvec{c}}^\top Z_{\hat{{\varvec{U}}}}^{-1} {\varvec{c}} + (1-r){\varvec{c}}^\top Z_{\hat{{\varvec{U}}}'}^{-1} {\varvec{c}}$ for any ${\varvec{c}}\in {\mathbb {R}}^S$, and thus $\big \Vert \big (rZ_{\hat{{\varvec{U}}}}+(1-r)Z_{\hat{{\varvec{U}}}'}\big )^{-1}\big \Vert _2 \le \Vert Z_{\hat{{\varvec{U}}}}^{-1}\Vert _2+\Vert Z_{\hat{{\varvec{U}}}'}^{-1}\Vert _2$. Therefore, we obtain

$$\begin{aligned} \Vert Z_{\hat{{\varvec{U}}}}^{-1}-Z_{\hat{{\varvec{U}}}'}^{-1}\Vert _{2} \le 2(\Vert Z_{\hat{{\varvec{U}}}}^{-1}\Vert _{{2}}^{2}+\Vert Z_{\hat{{\varvec{U}}}'}^{-1}\Vert _{{2}}^{2})\Vert Z_{\hat{{\varvec{U}}}}-Z_{\hat{{\varvec{U}}}'}\Vert _{{2}}. \end{aligned}$$

Now the statement follows. $\square$

As a consequence, we obtain the following.

### Lemma 3.6

Suppose that $\hat{{\varvec{U}}}\in [{\mathcal {H}}]^{S}$ is linearly independent and $\Vert \hat{{\varvec{U}}}\Vert _{[{\mathcal {H}}]^{S}}\le \alpha$ for some $\alpha >0$. Then, we have $\Vert Z_{\hat{{\varvec{U}}}}^{-1}\Vert \le \gamma$ for some $\gamma >0$. Further, there exists a constant $C_{\alpha ,S}>0$ that is independent of the position $\hat{{\varvec{U}}}$ and $R=R(\hat{{\varvec{U}}})\in (0,1]$ such that

$$\begin{aligned} \Vert Z_{\hat{{\varvec{V}}}}^{-1}-Z_{\hat{{\varvec{V}}}'}^{-1}\Vert \le \gamma ^{2}C_{\alpha ,S}\Vert \hat{{\varvec{V}}}-\hat{{\varvec{V}}}'\Vert _{[{\mathcal {H}}]^{S}} \end{aligned}$$

holds for any $\hat{{\varvec{V}}},\hat{{\varvec{V}}}'\in [{\mathcal {H}}]^{S}$, with $\Vert \hat{{\varvec{V}}}-\hat{{\varvec{U}}}\Vert _{[{\mathcal {H}}]^{S}}\le R$, $\Vert \hat{{\varvec{V}}}'-\hat{{\varvec{U}}}\Vert _{[{\mathcal {H}}]^{S}}\le R$.

### Proof

Since $\Vert Z_{\hat{{\varvec{U}}}}^{-1}\Vert _{{2}}\le \gamma$, for $R=R(\hat{{\varvec{U}}})\in (0,1]$ small enough we have $\Vert Z_{\hat{{\varvec{V}}}}^{-1}\Vert _{{2}}\le 2\gamma$ for all $\hat{{\varvec{V}}}\in [{\mathcal {H}}]^{S}$ such that $\Vert \hat{{\varvec{V}}}-\hat{{\varvec{U}}}\Vert _{[{\mathcal {H}}]^{S}}\le R$. Such $\hat{{\varvec{V}}}$ satisfies $\Vert \hat{{\varvec{V}}}\Vert _{[{\mathcal {H}}]^{S}}\le \alpha +R\le \alpha +1$. Thus, with ${\tilde{\alpha }}:=\alpha +1$ and $C_{\alpha ,S}:=8C_{{\tilde{\alpha }},S}$ in Lemma 3.5 the statement follows. $\square$

Lemmata 3.4 and 3.6 established above give the following local Lipschitz continuity of the non-linear term *G* we need.

### Proposition 3.7

Let Assumption 2 hold. Suppose that we have $\Vert Z_{{\hat{{\varvec{U}}}}}^{-1}\Vert _{{2}}\le \gamma$ for ${\hat{{\varvec{U}}}}\in [{\mathcal {H}}]^{S}$, and that ${\hat{{\varvec{Y}}}}\in [L^{2}(\varOmega )]^{S}$ is $L^{2}(\varOmega )$-orthonormal. Then, $G:{\mathcal {X}}\rightarrow {\mathcal {X}}$ defined in (3.1) satisfies the assumption of Proposition 3.1 for this $(\hat{{\varvec{U}}},\hat{{\varvec{Y}}})$ with a constant depending also on $\gamma$.

### Proof

Let $\alpha \ge \Vert \hat{{\varvec{U}}}\Vert _{[{\mathcal {H}}]^{S}}$ and $\beta \ge \Vert \hat{{\varvec{Y}}}\Vert _{[L^{2}(\varOmega )]^{S}}=\sqrt{S}$ be given. First, from Assumption 2 we have $\Vert [G_{1}(\hat{{\varvec{Y}}})](\hat{{\varvec{U}}})\Vert _{[{\mathcal {H}}]^{S}}\le {C_{\alpha ,\beta }}$, and further, together with $\Vert Z_{\hat{{\varvec{U}}}}^{-1}\Vert _{{2}}\le \gamma$ we have $\Vert [G_{2}(\hat{{\varvec{U}}})](\hat{{\varvec{Y}}})\Vert _{[{\mathcal {H}}]^{S}}\le {C_{\alpha ,\beta ,\gamma }}$. It now suffices to show

$$\begin{aligned}&\Vert \big [G_{1}({\varvec{W}})\big ]({\varvec{V}})-\big [G_{1}\big ({\varvec{W}}'\big )\big ]({\varvec{V}}')\Vert _{[{\mathcal {H}}]^{S}}\\&\quad \le C_{\alpha ,\beta }\Big (\Vert {\varvec{V}}-{{\varvec{V}}}'\Vert _{[{\mathcal {H}}]^{S}}^{2}+\Vert {\varvec{W}}-{\varvec{W}}'\Vert _{[L^{2}(\varOmega )]^{S}}^{2}\Big )^{1/2}, \end{aligned}$$

and

$$\begin{aligned}&\Vert \big [G_{2}({\varvec{V}})\big ]({\varvec{W}})-\big [G_{2}({\varvec{V}}')\big ]({\varvec{W}}')\Vert _{[L^{2}(\varOmega )]^{S}}\\&\quad \le C'_{\alpha ,\beta ,\gamma }\Big (\Vert {\varvec{V}}-{\varvec{V}}'\Vert _{[{\mathcal {H}}]^{S}}^{2}+\Vert {\varvec{W}}-{\varvec{W}}'\Vert _{[L^{2}(\varOmega )]^{S}}^{2}\Big )^{1/2} \end{aligned}$$

in closed balls centred at $\hat{{\varvec{U}}}$, and $\hat{{\varvec{Y}}}$, respectively, with a radius ${r=r(\hat{{\varvec{U}}},\hat{{\varvec{Y}}})}$. The first inequality can be checked from Assumption 2. The second inequality follows from Lemmata 3.4 and 3.6 by letting $r<\min \{R(\hat{{\varvec{U}}}),{\overline{\kappa }}(1,\beta )\}$, where ${\overline{\kappa }}(1,\beta )$ is as in Lemma 3.3. $\square$

## Existence and regularity

We will now show the existence of the Dual DO solution on the maximal interval. We start with local existence of the mild solution

### Proposition 4.1

(Mild, local) Let Assumptions 1 and 2 hold. Suppose that the initial condition ${\varvec{U}}_{0}\in [{\mathcal {H}}]^{S}$ is linearly independent in ${\mathcal {H}}$, and ${\varvec{Y}}_{0}\in [L^{2}(\varOmega )]^{S}$ is orthonormal in $L^{2}(\varOmega )$. Then, there exists $t^{*}{=t^{*}({\varvec{U}}_{0},{\varvec{Y}}_{0})}>0$ such that the mild solution of the abstract Cauchy problem (3.2) uniquely exists on $[0,t^{*}]$.

### Proof

In view of Proposition 3.7, the statement follows from Proposition 3.1. $\square$

A regularity of the initial condition gives us the existence of the strong solution.

### Proposition 4.2

(Strong, local) Let Assumptions 1 and 2 hold. Suppose further that the initial condition ${\varvec{U}}_{0}\in [{\mathcal {H}}]^{S}$ is linearly independent in ${\mathcal {H}}$, and ${\varvec{Y}}_{0}\in [L^{2}(\varOmega )]^{S}$ is orthonormal in $L^{2}(\varOmega )$. Furthermore, suppose that $({\varvec{U}}_{0},{\varvec{Y}}_{0})\in D(A)$. Then, the mild solution obtained in Proposition 4.1 is the strong solution of the abstract Cauchy problem (3.2).

### Proof

In view of [18, Theorem 6.1.6], the statement follows from Proposition 4.1. $\square$

The above solution is actually the Dual DO solution.

### Corollary 4.3

(Dual DO-strong, local) Let the assumptions of Proposition 4.2 hold. Then, the strong solution $({\varvec{U}}(t),{\varvec{Y}}(t))$ of the abstract Cauchy problem (3.2) uniquely exists on a non-empty interval $[0,t^{*}]$. The solution ${\varvec{U}}(t)$ stays linearly independent on $[0,t^{*}]$ and the solution ${\varvec{Y}}(t)$ is orthonormal in $L^{2}(\varOmega )$ for $t\in [0,t^{*}]$ and satisfies the gauge condition ${\mathbb {E}}[\dot{{\varvec{Y}}}(t) {\varvec{Y}}(t)^\top ]=0$ for almost every $t\in [0,t^*]$. Hence, the Dual DO solution uniquely exists in the strong sense on $[0,t^{*}]$.

### Proof

It suffices to show the linear independence of ${\varvec{U}}(t)$ and the orthonormality of ${\varvec{Y}}(t)$. But the solution of the abstract Cauchy problem (3.2) established in Proposition 4.2 exists only on an interval $[0,t^{*}]$ on which the inverse Gram matrix $Z_{{\varvec{U}}}^{-1}$ is well defined. Hence, on this interval, ${\varvec{U}}(t)$ is linear independent.

To see the orthonormality, first note that, from the absolute continuity of ${\varvec{Y}}(t)$, the function ${\mathbb {E}}[Y_{j}Y_{k}]$ is absolutely continuous on [0, *T*]. But following the same argument as (2.7), we have $\frac{\mathrm {d}}{\mathrm {d}t}{\mathbb {E}}[Y_{j}Y_{k}]= {\mathbb {E}}[{\dot{Y}}_{j}Y_{k}]+{\mathbb {E}}[Y_{j}{\dot{Y}}_{k}]=0$ a.e. on [0, *T*]. Therefore, from the orthonormality of the initial condition, for every $t\in [0,t^{*}]$ we have ${\mathbb {E}}[Y_{j}(t)Y_{k}(t)]-\delta _{jk}=\int _0^t0\mathrm {d}t=0$, where $\delta _{jk}=1$ only if $j=k$, and 0 otherwise. Hence, ${\varvec{Y}}(t)$ is orthonormal for all $t\in [0,t^*]$. $\square$

With a further regularity of *F*, we obtain the classical Dual DO solution.

### Corollary 4.4

(Dual DO-classical, local) Suppose that Assumptions 1, 2 and 3 are satisfied. Suppose further that the initial condition ${\varvec{U}}_{0}\in D(\varLambda )$ is linearly independent in ${\mathcal {H}}$, and ${\varvec{Y}}_{0}\in [L^{2}(\varOmega )]^{S}$ is orthonormal in $L^{2}(\varOmega )$. Then, there exists $t^{*}>0$ such that the Dual DO solution uniquely exists in the classical sense on $[0,t^{*}]$.

### Proof

We first observe that $G:[{\mathcal {V}}]^{S}\oplus [L^{2}(\varOmega )]^{S}\rightarrow [{\mathcal {V}}]^{S}\oplus [L^{2}(\varOmega )]^{S}$ is locally Lipschitz, where ${\mathcal {V}}$ is the Hilbert space $D_{{\mathcal {H}}}(\varLambda )$ equipped with the graph norm. Further, we note that $(\mathrm {e}^{tA})_{t\ge 0}$ is a $C_{0}$ semigroup on $[{\mathcal {V}}]^{S}\oplus [L^{2}(\varOmega )]^{S}$.

With these in mind, we see that a result analogous to Proposition 3.1 holds in $[{\mathcal {V}}]^{S}\oplus [L^{2}(\varOmega )]^{S}$. Then, in view of the discussion in [18, pages 190–191], the statement follows from the similar argument as in the proof of Corollary 4.3. $\square$

We now extend the solution to the maximal time interval.

We start with the following bound.

### Lemma 4.5

Let Assumption 4 hold. Suppose that the strong solution $({\varvec{U}}(t),{\varvec{Y}}(t))$ of the abstract Cauchy problem (3.2) exists on $[0,t^{*}]$. Then, we have $\Vert {\varvec{Y}}(t)\Vert _{[L^{2}(\varOmega )]^{S}}=\Vert {\varvec{Y}}_{0}\Vert _{[L^{2}(\varOmega )]^{S}}$ for all $t\in [0,t^{*}]$. Furthermore, we have

$$\begin{aligned} \Vert {\varvec{U}}(t)\Vert _{[{\mathcal {H}}]^{S}}\le (\sqrt{2C_{\varLambda ,F}}t^{1/2}+\Vert {\varvec{U}}_{0}\Vert _{[{\mathcal {H}}]^{S}}\mathrm {e}^{C_{\varLambda ,F}t})\quad \text { for all }t\in [0,t^{*}]. \end{aligned}$$

### Proof

Following the same argument as (2.7), we have ${\mathbb {E}}\big [Y_{j}\frac{\partial }{\partial t}Y_{j}\big ]=0$ a.e. $[0,t^{*}]$, $j=1,\dots ,S$. Hence, $\Vert {\varvec{Y}}(t)\Vert _{[L^{2}(\varOmega )]^{S}}$ is constant a.e. $[0,t^{*}]$. Then, the continuity of $t\mapsto \Vert {\varvec{Y}}(t)\Vert _{[L^{2}(\varOmega )]^{S}}$ implies the first statement. Next, a.e. in $[0,t^{*}]$ we have

$$\begin{aligned} \frac{\partial }{\partial t}{\varvec{U}}^{\top } = \varLambda ({\varvec{U}}^{\top }) +{\mathbb {E}}\big [F(u_{S}){\varvec{Y}}^{\top }\big ] = {\mathbb {E}}\big [\varLambda ({\varvec{U}}^{\top }{\varvec{Y}}){\varvec{Y}}^{\top }\big ] +{\mathbb {E}}\big [F(u_{S}){\varvec{Y}}^{\top }\big ], \end{aligned}$$

where each component is in ${\mathcal {H}}$, and hence for $j=1,\dots ,S$, we have $\langle \frac{\partial }{\partial t}U_{j},U_{j}\rangle = {\mathbb {E}}[\langle \varLambda (u_{S})],U_{j}Y_{j}\rangle ]+{\mathbb {E}}[\langle F(u_{S}),U_{j}Y_{j}\rangle ]$. Hence, because of Assumption 4 and the orthonormality of $\{Y_{j}\}$ we have

$$\begin{aligned} \frac{\mathrm {d}}{\mathrm {d}t}\sum _{j=1}^{S}\Vert U_{j}\Vert ^{2} =2{\mathbb {E}}[\langle \varLambda (u_{S})+F(u_{S}),u_{S}\rangle ] \le 2C_{\varLambda ,F}\Bigl (1+\sum _{j=1}^{S}\Vert U_{j}\Vert ^{2}\Bigr ), \end{aligned}$$

and thus $\displaystyle {\sum _{j=1}^{S}\Vert U_{j}(t)\Vert ^{2}\le (2C_{\varLambda ,F}t+\sum _{j=1}^{S}\Vert U_{j}(0)\Vert ^{2})+2C_{\varLambda ,F}\int _{0}^{t}\sum _{j=1}^{S}\Vert U_{j}(s)\Vert ^{2}\mathrm {d}s}$. Therefore, the Gronwall’s inequality implies that the second statement holds for almost every *t*. Noting that the mapping $t\mapsto [\Vert {\varvec{U}}(t)\Vert _{[{\mathcal {H}}]^{S}}-(\sqrt{2C_{\varLambda ,F}}t^{1/2}+\Vert {\varvec{U}}(0)\Vert _{[{\mathcal {H}}]^{S}})\mathrm {e}^{C_{\varLambda ,F}t}]$ is continuous, this is true for every $t\in [0,t^{*}]$. $\square$

We are ready to establish the existence of a Dual DO solution until ${\varvec{U}}$ becomes linearly dependent.

### Theorem 4.6

(Dual DO-strong, maximal) Suppose that Assumptions 1, 2, and 4 are satisfied, and that the initial condition ${\varvec{U}}_{0}\in [{\mathcal {H}}]^{S}$ is linearly independent in ${\mathcal {H}}$, and ${\varvec{Y}}_{0}\in [L^{2}(\varOmega )]^{S}$ is orthonormal in $L^{2}(\varOmega )$. Further, suppose that $({\varvec{U}}_{0},{\varvec{Y}}_{0})\in D(A)$. Then, there exists $t_{\max }>0$ such that the Dual DO solution uniquely exists in the strong sense on $[0,t_{\max })$. The solution can be extended in time until the Gram matrix $Z_{{\varvec{U}}}$ of ${\varvec{U}}$ becomes singular: we have either

$$\begin{aligned} t_{\max }=\infty ,\quad \text {or}\quad \lim _{t\uparrow t_{\max }}\Vert Z_{{\varvec{U}}(t)}^{-1}\Vert _{{2}}=\infty . \end{aligned}$$

### Proof

Under the condition $({\varvec{U}}_{0},{\varvec{Y}}_{0})\in D(A)$, it suffices to show the maximality of the mild solution. We show that $t_{\max }<\infty$ implies $\lim _{t\uparrow t_{\max }}\Vert Z_{{\varvec{U}}(t)}^{-1}\Vert _{{2}}=\infty$. In this regard, we first show $\limsup _{t\uparrow t_{\max }}\Vert Z_{{\varvec{U}}(t)}^{-1}\Vert _{{2}}=\infty$. We argue by contradiction and assume $t_{\max }<\infty$ and $\limsup _{t\uparrow t_{\max }}\Vert Z_{{\varvec{U}}(t)}^{-1}\Vert _{{2}}<\infty$. Then we have

$$\begin{aligned} \sup _{t\in [t_{\max }-\delta ,t_{\max })}\Vert Z_{{\varvec{U}}(t)}^{-1}\Vert _{{2}}<\infty \quad \text { for sufficiently small }\delta >0. \end{aligned}$$

Thus, since $\max _{t\in [0,t_{\max }-\delta ]}\Vert Z_{{\varvec{U}}(t)}^{-1}\Vert _{{2}}<\infty$ for any $0<\delta <t_{\max }$, with a constant $K>0$ we have $\Vert Z_{{\varvec{U}}(t)}^{-1}\Vert _{{2}}<K$ for all $t\in [0,t_{\max })$. Now Lemma 4.5 implies $\Vert {\varvec{Y}}(t)\Vert _{[L^{2}(\varOmega )]^{S}}=\sqrt{S}$ and

$$\begin{aligned} \Vert {\varvec{U}}(t)\Vert _{[{\mathcal {H}}]^{S}}\le \alpha _{\max }:=(\sqrt{2C_{\varLambda ,F}}t_{\max }^{1/2}+\Vert {\varvec{U}}_{0}\Vert _{[{\mathcal {H}}]^{S}}\mathrm {e}^{C_{\varLambda ,F}t_{\max }}),\quad t\in [0,t_{\max }), \end{aligned}$$

and thus in view of Proposition 3.7 we have $\Vert [G_{1}({\varvec{Y}}(s))]({\varvec{U}}(s))\Vert _{[{\mathcal {H}}]^{S}}\le C_{\alpha _{\max },S}$ for any $s\in [0,t_{\max })$. If $0<t<t'<t_{\max }$ then letting $K_{\varLambda }=\sup _{r\in [0,t_{\max }]}\Vert \mathrm {e}^{r\varLambda }\Vert$ we have

$$\begin{aligned}&\Vert {\varvec{U}}(t')-{\varvec{U}}(t)\Vert _{[{\mathcal {H}}]^{S}} \\&\quad \le \Vert \mathrm {e}^{t'\varLambda }{\varvec{U}}(0)-\mathrm {e}^{t\varLambda }{\varvec{U}}(0)\Vert _{[{\mathcal {H}}]^{S}}+(t'-t)K_{\varLambda }C_{\alpha _{\max },S}\\&\qquad +\int _{0}^{t_{\max }}\Vert \mathrm {e}^{(t-s)\varLambda }\Vert \Vert (\mathrm {e}^{(t'-t)\varLambda }-I)[G_{1}({\varvec{Y}}(s))]({\varvec{U}}(s))\Vert _{[{\mathcal {H}}]^{S}}\mathrm {d}s. \end{aligned}$$

From $\Vert [G_{1}({\varvec{Y}}(s))]({\varvec{U}}(s))\Vert _{[{\mathcal {H}}]^{S}}\le C_{\alpha _{\max },S}$, the dominated convergence theorem implies that the right hand side of

$$\begin{aligned}&\int _{0}^{t_{\max }}\Vert \mathrm {e}^{(t-s)\varLambda }\Vert \Vert (\mathrm {e}^{(t'-t)\varLambda }-I)[G_{1}({\varvec{Y}}(s))]({\varvec{U}}(s))\Vert _{[{\mathcal {H}}]^{S}}\mathrm {d}s\\&\quad \le \int _{0}^{t_{\max }}K_{\varLambda } \Vert (\mathrm {e}^{(t'-t)\varLambda }-I)[G_{1}({\varvec{Y}}(s))]({\varvec{U}}(s)) \Vert _{[{\mathcal {H}}]^{S}}\mathrm {d}s \end{aligned}$$

tends to zero as $t,t'$ tend to $t_{\max }$. Hence, $\Vert {\varvec{U}}(t')-{\varvec{U}}(t)\Vert _{[{\mathcal {H}}]^{S}}\rightarrow 0$ as $t,t'\rightarrow t_{\max }$. Therefore, ${\varvec{U}}$ admits a continuous extension $\lim _{t\uparrow t_{\max }}{\varvec{U}}(t)={\varvec{U}}(t_{\max })$. This allows us to extend $Z_{{\varvec{U}}(t)}^{-1}$ to $[0,t_{\max }]$. Indeed, Lemma 3.5 implies

$$\begin{aligned} \Vert Z_{{\varvec{U}}(t')}^{-1}-Z_{{\varvec{U}}(t)}^{-1}\Vert _{{2}}\le 2C_{\alpha _{\max },S}{K^{2}}\Vert {\varvec{U}}(t')-{\varvec{U}}(t)\Vert _{[{\mathcal {H}}]^{S}}, \end{aligned}$$

and thus we have $\lim _{t\uparrow t_{\max }}Z_{{\varvec{U}}(t)}^{-1}={Z^{*}\in {\mathbb {R}}^{S\times S}}$ with ${\Vert Z^{*}\Vert _{{2}}\le K}$, but we must have $Z^{*}=Z_{{\varvec{U}}(t_{\max })}^{-1}$. Similarly, noting that $\Vert Z_{{\varvec{U}}(s)}^{-1}\Vert _{{2}}<K$ implies

$$\begin{aligned} \Vert [G_{2}({\varvec{Y}}(s))]({\varvec{U}}(s))\Vert _{[L^{2}(\varOmega )]^{S}}\le C_{\alpha _{\max },S,K}, \end{aligned}$$

we see that $\lim _{t\uparrow t_{\max }}{\varvec{Y}}(t)={\varvec{Y}}(t_{\max })$ exists, and from Corollary 4.3 we have ${\mathbb {E}}[{\varvec{Y}}(t_{\max }){\varvec{Y}}(t_{\max })^{\top }]=I$. But in view of Proposition 3.7 these consequences imply that we can extend the solution beyond $t_{\max }$, which contradicts the maximality of $[0,t_{\max })$. Hence, $\limsup _{t\uparrow t_{\max }}\Vert Z_{{\varvec{U}}(t)}^{-1}\Vert _{{2}}=\infty$.

To conclude the proof we will show

$$\begin{aligned} \lim _{t\uparrow t_{\max }}\Vert Z_{{\varvec{U}}(t)}^{-1}\Vert _{{2}}=\infty . \end{aligned}$$

If this is false, then there exist a sequence $t_{n}\uparrow t_{\max }$ and $\gamma >0$ such that $\Vert Z_{{\varvec{U}}(t_{n})}^{-1}\Vert _{{2}}\le \gamma$ for all $n\ge 0$. But since $\limsup _{t\uparrow t_{\max }}\Vert Z_{{\varvec{U}}(t)}^{-1}\Vert _{{2}}=\infty$ there is a sequence $s_{k}\uparrow t_{\max }$ such that $\Vert Z_{{\varvec{U}}(s_{k})}^{-1}\Vert _{{2}}\ge \gamma +1$ for all $k\ge 0$. We take a subsequence $(s_{k_{n}})_{n}$ so that $t_{n}<s_{k_{n}}$ for all *n*. From the continuity of $t\mapsto \Vert Z_{{\varvec{U}}(t)}^{-1}\Vert _{{2}}$ on $[t_{n},s_{k_{n}}]$, there exists $h_{n}\in [0,s_{k_{n}}-t_{n}]$ such that $\Vert Z_{{\varvec{U}}(t_{n}+h_{n})}^{-1}\Vert _{{2}}=\gamma +1$. Now, from Lemma 3.5 we have for any $n\ge 0$

$$\begin{aligned} 1 \!\le \! \Vert Z_{{\varvec{U}}(t_{n}+h_{n})}^{-1}\!\!\;\Vert _{{2}}-\Vert Z_{{\varvec{U}}(t_{n})}^{-1}\Vert _{{2}} \!\le \! C_{\alpha _{\max },S}{(2\gamma ^{2}\!\!\;+2\gamma +1)}\Vert {\varvec{U}}(t_{n}+h_{n})-{\varvec{U}}(t_{n})\Vert _{\!\!\;[{\mathcal {H}}]^{S}}, \end{aligned}$$

which is absurd since $|h_{n}|\le |s_{k_{n}}-t_{\max }|+|t_{\max }-t_{n}|\rightarrow 0$ as $n\rightarrow \infty$ and ${\varvec{U}}$ is continuous on $[0,t_{\max })$. Hence, the proof is complete. $\square$

Under a stronger assumption on the non-linear term *F*, we obtain the following bound for $\Vert \varLambda {\varvec{U}}(t)\Vert _{[{\mathcal {H}}]^{S}}$. This bound will be used to establish the existence in the classical sense on the maximal interval.

### Lemma 4.7

Let Assumptions 1 and 5 hold. Suppose that the classical solution $({\varvec{U}}(t),{\varvec{Y}}(t))$ of the abstract Cauchy problem (3.2) exists on $[0,t^{*}]$ for some $t^{*}>0$. Then, we have

$$\begin{aligned} \Vert \varLambda {\varvec{U}}(t)\Vert _{[{\mathcal {H}}]^{S}}\le {K_{\varLambda }}(\Vert \varLambda {\varvec{U}}(0)\Vert _{[{\mathcal {H}}]^{S}}+tC_{F}\sqrt{S})\mathrm {e}^{{K_{\varLambda }}C_{F}t}\quad \text { for }t\in [0,t^{*}], \end{aligned}$$

where the constant $C_{F}>0$ is from Assumption 5.

### Proof

We have

$$\begin{aligned} \varLambda {\varvec{U}}(t)&=\varLambda e^{t\varLambda }{\varvec{U}}(0)+\int _{0}^{t}\varLambda e^{(t-\tau )\varLambda }{\mathbb {E}}[F({\varvec{U}}(\tau )^{\top }{\varvec{Y}}(\tau )){\varvec{Y}}(\tau )]\mathrm {d}\tau \\ {}&=e^{t\varLambda }\varLambda {\varvec{U}}(0)+\int _{0}^{t}e^{(t-\tau )\varLambda }{\mathbb {E}}[\varLambda F({\varvec{U}}(\tau )^{\top }{\varvec{Y}}(\tau )){\varvec{Y}}(\tau )]\mathrm {d}\tau , \end{aligned}$$

and thus, as Assumption 1 implies $\Vert e^{s\varLambda }\Vert _{[{\mathcal {H}}]^{S}\rightarrow [{\mathcal {H}}]^{S}}\le {K_{\varLambda }}$, $s\ge 0$, we get $\Vert \varLambda {\varvec{U}}(t)\Vert _{[{\mathcal {H}}]^{S}}\le {K_{\varLambda }}\Vert \varLambda {\varvec{U}}(0)\Vert _{[{\mathcal {H}}]^{S}}+{K_{\varLambda }}\int _{0}^{t}{\mathbb {E}}[\Vert \varLambda F({\varvec{U}}(\tau )^{\top }{\varvec{Y}}(\tau )){\varvec{Y}}(\tau )\Vert _{[{\mathcal {H}}]^{S}}]\mathrm {d}\tau$. From ${\mathbb {E}}[|{\varvec{Y}}(\tau )|^{2}]={\varvec{1}}$ and Assumption 5, we have

$$\begin{aligned} {\mathbb {E}}[\Vert \varLambda F({\varvec{U}}(\tau )^{\top }{\varvec{Y}}(\tau )){\varvec{Y}}(\tau )\Vert _{[{\mathcal {H}}]^{S}}]&\le C_{F}(1+\Vert \varLambda {\varvec{U}}(\tau )\Vert _{[{\mathcal {H}}]^{S}})\sqrt{S}, \end{aligned}$$

hence $\Vert \varLambda {\varvec{U}}(t)\Vert _{[{\mathcal {H}}]^{S}}\le {K_{\varLambda }}(\Vert \varLambda {\varvec{U}}(0)\Vert _{[{\mathcal {H}}]^{S}}+tC_{F}\sqrt{S})+{K_{\varLambda }}C_{F}\int _{0}^{t}\Vert \varLambda {\varvec{U}}(\tau )\Vert _{[{\mathcal {H}}]^{S}}\mathrm {d}\tau$. Then, applying the Gronwall’s inequality completes the proof. $\square$

### Theorem 4.8

(Dual DO-classical, maximal) Suppose that Assumptions 1–5 are satisfied. Suppose further that the initial condition ${\varvec{U}}_{0}\in [{\mathcal {H}}]^{S}$ is linearly independent in ${\mathcal {H}}$, and ${\varvec{Y}}_{0}\in [L^{2}(\varOmega )]^{S}$ is orthonormal in $L^{2}(\varOmega )$. Further, suppose that $({\varvec{U}}_{0},{\varvec{Y}}_{0})\in D(A)$. Then, there exists $t_{\max }>0$ such that the Dual DO solution uniquely exists in the classical sense on $[0,t_{\max })$. The solution can be extended in time until the Gram matrix $Z_{{\varvec{U}}}$ of ${\varvec{U}}$ becomes singular: we have either

$$\begin{aligned} t_{\max }=\infty ,\quad \text {or}\quad \lim _{t\uparrow t_{\max }}\Vert Z_{{\varvec{U}}(t)}^{-1}\Vert _{{2}}=\infty . \end{aligned}$$

### Proof

Our argument is analogous to the proof of Theorem 4.6, but here we consider the parameter equation in $[{\mathcal {V}}]^{S}\oplus [L^{2}(\varOmega )]^{S}$. The only difference thus is the equation for ${\varvec{U}}$, but Lemmata 4.5 and 4.7 give a bound for $\Vert {\varvec{U}}(t)\Vert _{[{\mathcal {V}}]^{S}}$, $t\in [0,t_{\max })$, and Assumption 5 gives a bound for $\Vert [G_{1}({\varvec{Y}}(s))]({\varvec{U}}(s))\Vert _{[{\mathcal {V}}]^{S}}$, $s\in [0,t_{\max })$. Further, we have

$$\begin{aligned} \sup _{r\in [0,t_{\max }]}\Vert \mathrm {e}^{r\varLambda }\Vert _{[{\mathcal {V}}]^{S}\rightarrow [{\mathcal {V}}]^{S}}\le \sup _{r\in [0,t_{\max }]}\Vert \mathrm {e}^{r\varLambda }\Vert _{[{\mathcal {H}}]^{S}\rightarrow [{\mathcal {H}}]^{S}}\le K_{\varLambda }, \end{aligned}$$

and $\Vert \mathrm {e}^{s\varLambda }-\mathrm {e}^{t\varLambda }\Vert _{[{\mathcal {V}}]^{S}\rightarrow [{\mathcal {V}}]^{S}}\le \Vert \mathrm {e}^{s\varLambda }-\mathrm {e}^{t\varLambda }\Vert _{[{\mathcal {H}}]^{S}\rightarrow [{\mathcal {H}}]^{S}}$. Noting that $({\varvec{U}},{\varvec{Y}})$ is also a mild solution in ${\mathcal {X}}$, the extension of $Z_{{\varvec{U}}}^{-1}$ can be established. Hence, we see that the mild solution in $[{\mathcal {V}}]^{S}\oplus [L^{2}(\varOmega )]^{S}$ exists on $[0,t_{\max })$, and that if $t_{\max }<\infty$ then $\lim _{t\uparrow t_{\max }}\Vert Z_{{\varvec{U}}(t)}^{-1}\Vert _{{2}}=\infty$. But in view of [18, Corollary 4.2.6, Theorem 6.1.7] this is a classical solution, and thus the proof is complete. $\square$

We are now interested in continuing the DLR approximation $u_{S}$ beyond the maximal time $t_{\max }$. A difficulty arising is the full rank condition imposed on $M_{S}$: at $t_{\max }$ the spatial basis becomes linearly dependent, and thus the solution will not stay in $M_{S}$. But from a practical point of view this should be favourable—roughly speaking, at the maximal time a smaller basis is sufficient to capture the same information as ${\varvec{U}}$ does. This observation motivates us to leave $M_{S}$: to extend the approximation beyond $t_{\max }$ we consider the extension to $t_{\max }$ in the ambient space $L^{2}(\varOmega ;{\mathcal {H}})$. To do so, we go back to the original formulation (2.4). Then, upon extending the solution to $t_{\max }$, one can re-start from $t_{\max }$ with a suitable decomposition as the initial condition.

### Proposition 4.9

Let the assumptions of Theorem 4.8 hold. Then, with the classical solution $({\varvec{U}}\!\!\;,{\varvec{Y}})$ as in Theorem 4.8, $u_{S}={\varvec{U}}^{\!\!\;\top }{\varvec{Y}}:[0,t_{\max })\rightarrow L^{2}(\varOmega ;{\mathcal {H}})$ is Lipschitz continuous. Thus, $u_{S}$ admits a unique continuous extension to $[0,t_{\max }]$.

### Proof

Noting that $u_S$ is absolutely continuous on $[0,t]\subset [t_{\max })$, for any $0\le t'<t<t_{\max }$ we have $\Vert u_{S}(t)-u_{S}(t')\Vert _{L^{2}(\varOmega ;{\mathcal {H}})}\le \int _{t'}^{t}\big (\Vert \varLambda u_{S}(r)\Vert _{L^{2}(\varOmega ;{\mathcal {H}})}+\Vert F(u_{S}(r))\Vert _{L^{2}(\varOmega ;{\mathcal {H}})}\big )\mathrm {d}r$. But from Lemma 4.7 and Assumption 2 we have

$$\begin{aligned} \Vert \varLambda u_{S}(r)\Vert _{L^{2}(\varOmega ;{\mathcal {H}})}\le \sum _{j=1}^{S}\Vert \varLambda U_{j}(r)\Vert \le \sqrt{S}(K_{\varLambda }\Vert \varLambda {\varvec{U}}(0)\Vert _{[{\mathcal {H}}]^{S}}+t_{\max }C_{F}\sqrt{S}), \end{aligned}$$

and $\Vert F(u_{S}(r))\Vert _{L^{2}(\varOmega ;{\mathcal {H}})}\le C_{t_{\max },K_{\varLambda },F}$ for some constant $C_{t_{\max },K_{\varLambda },F}\!>0$. Hence, we obtain

$$\begin{aligned} \Vert u_{S}(t)&-u_{S}(t')\Vert _{L^{2}(\varOmega ;{\mathcal {H}})}\\&\le (t-t')\big (\sqrt{S}(K_{\varLambda }\Vert \varLambda {\varvec{U}}(0)\Vert _{[{\mathcal {H}}]^{S}}+t_{\max }C_{F}\sqrt{S})+C_{t_{\max },K_{\varLambda },F}\big ), \end{aligned}$$

and thus $u_{S}$ admits a continuous extension $u_{S}(t)\rightarrow u^{*}=:u_{S}(t_{\max })$ as $t\uparrow t_{\max }$. $\square$

### Proof of Proposition 2.2

Finally, we will show the existence of a smooth parametrisation given a smooth curve $[0,T]\ni t\mapsto u_S(t)\in M_S$, announced in Proposition 2.2. Our argument is similar to the existence proofs in this section thus far.

#### Proof of Proposition 2.2

Consider the following ordinary differential equation in ${\mathbb {R}}^{S\times S}\oplus [{\mathcal {H}}]^{S}\oplus [L^{2}(\varOmega )]^{S}$:

$$\begin{aligned} {\dot{\varSigma }}&={\mathbb {E}}[\langle \tilde{{\varvec{V}}},{\dot{u}}_{S}{\varvec{W}}^{\top }\rangle ]\\ \dot{\tilde{{\varvec{V}}}}^{\top }\varSigma&={\mathbb {E}}[{\dot{u}}_{S}{\varvec{W}}^{\top }]-\tilde{{\varvec{V}}}^{\top }\langle \tilde{{\varvec{V}}}{\mathbb {E}}[{\dot{u}}_{S}{\varvec{W}}^{\top }]\rangle =:(I-P_{\tilde{{\varvec{V}}}})\big ({\mathbb {E}}[{\dot{u}}_{S}{\varvec{W}}^{\top }]\big )\\ \varSigma \dot{{\varvec{W}}}&=\langle \tilde{{\varvec{V}}},{\dot{u}}_{S}\rangle -{\mathbb {E}}[\langle \tilde{{\varvec{V}}},{\dot{u}}_{S}\rangle {\varvec{W}}^{\top }]{\varvec{W}}=:(I-P_{{\varvec{W}}})\langle \tilde{{\varvec{V}}},{\dot{u}}_{S}\rangle . \end{aligned}$$

If this equation has a solution $(\varSigma ,\tilde{{\varvec{V}}},{\varvec{W}})$ with the desired smoothness, then the statement follows.

But from ${\dot{u}}_{S}\in L^{1}([0,T];L^{2}(\varOmega ;{\mathcal {H}}))$ and the local Lipschitz continuity of the projection-operator-valued mappings, see Lemma 3.4, there exists a unique solution locally in time. Moreover, any solution $\tilde{{\varvec{V}}}$ and ${\varvec{W}}$ must preserve the orthogonality, see the proof of Corollary 4.3. Furthermore, Lemma 2.1 guarantees the stability and the invertibility of $\varSigma$ on [0, *T*]. Thus, following an argument similar to that of the proof of Theorem 4.6, we observe that the solution $(\varSigma ,\tilde{{\varvec{V}}},{\varvec{W}})$ can be uniquely extended to [0, *T*]. Now the proof is complete. The proof for the continuous differentiability is analogous. $\square$

## Conclusions

We established the existence of the dynamical low rank (DLR) approximation for random semi-linear evolutionary equations on the maximal interval. A key was to consider an equivalent formulation, the Dual DO formulation. After showing that the Dual DO formulation is indeed equivalent, we showed the unique existence of the solution in the strong and classical sense, by invoking results for the abstract Cauchy problem in the vector spaces. Further, we considered a continuation of the DLR approximation beyond the maximal time interval.
